# Analysis of microplastics in drinking water and other clean water samples with micro-Raman and micro-infrared spectroscopy: minimum requirements and best practice guidelines

**DOI:** 10.1007/s00216-021-03498-y

**Published:** 2021-07-20

**Authors:** Darena Schymanski, Barbara E. Oßmann, Nizar Benismail, Kada Boukerma, Gerald Dallmann, Elisabeth von der Esch, Dieter Fischer, Franziska Fischer, Douglas Gilliland, Karl Glas, Thomas Hofmann, Andrea Käppler, Sílvia Lacorte, Julie Marco, Maria EL Rakwe, Jana Weisser, Cordula Witzig, Nicole Zumbülte, Natalia P. Ivleva

**Affiliations:** 1 Chemical and Veterinary Analytical Institute Münsterland-Emscher-Lippe (CVUA-MEL), Joseph-König-Straße 40, 48147 Münster, Germany; 2 Institute of Food Chemistry, Westfälische Wilhelms-Universität Münster, Corrensstr. 45, 48149 Münster, Germany; 3 Bavarian Health and Food Safety Authority, Eggenreuther Weg 43, 91058 Erlangen, Germany; 4 Nestle Quality Assurance Center Vittel, 1020 Avenue Georges Clemenceau, 88800 Vittel, France; 5 Ifremer, REM/RDT/LDCM, 29280 Plouzané, France; 6 SGS Institut Fresenius GmbH, Königsbrücker Landstr. 161, 01109 Dresden, Germany; 7 Institute of Hydrochemistry, Chair of Analytical Chemistry and Water Chemistry, Department of Chemistry, Technical University of Munich, Elisabeth-Winterhalter-Weg 6, 81377 Munich, Germany; 8 Leibniz Institute of Polymer Research Dresden (IPF), Hohe Straße 6, 01069 Dresden, Germany; 9 Joint Research Centre (JRC), European Commission, 21027 Ispra, Italy; 10 Chair of Food Chemistry and Molecular Sensory Science, Technical University of Munich, Lise-Meitner-Straße 34, 85354 Freising, Germany; 11 Department of Environmental Chemistry, IDAEA-CSIC, Jordi Girona 18-26, 08034 Barcelona, Catalonia Spain; 12 Danone Waters, 11 Avenue du Général Dupas, 74500 Evian les Bains, France; 13 TZW: DVGW-Technologiezentrum Wasser (German Water Centre), Karlsruher Straße 84, 76139 Karlsruhe, Germany

**Keywords:** Microplastic, Micro-Raman spectroscopy, Micro-(FT)IR spectroscopy, Bottled water, Drinking water, Clean water

## Abstract

Microplastics are a widespread contaminant found not only in various natural habitats but also in drinking waters. With spectroscopic methods, the polymer type, number, size, and size distribution as well as the shape of microplastic particles in waters can be determined, which is of great relevance to toxicological studies. Methods used in studies so far show a huge diversity regarding experimental setups and often a lack of certain quality assurance aspects. To overcome these problems, this critical review and consensus paper of 12 European analytical laboratories and institutions, dealing with microplastic particle identification and quantification with spectroscopic methods, gives guidance toward harmonized microplastic particle analysis in clean waters. The aims of this paper are to (i) improve the reliability of microplastic analysis, (ii) facilitate and improve the planning of sample preparation and microplastic detection, and (iii) provide a better understanding regarding the evaluation of already existing studies. With these aims, we hope to make an important step toward harmonization of microplastic particle analysis in clean water samples and, thus, allow the comparability of results obtained in different studies by using similar or harmonized methods. Clean water samples, for the purpose of this paper, are considered to comprise all water samples with low matrix content, in particular drinking, tap, and bottled water, but also other water types such as clean freshwater.

**Graphical abstract:**

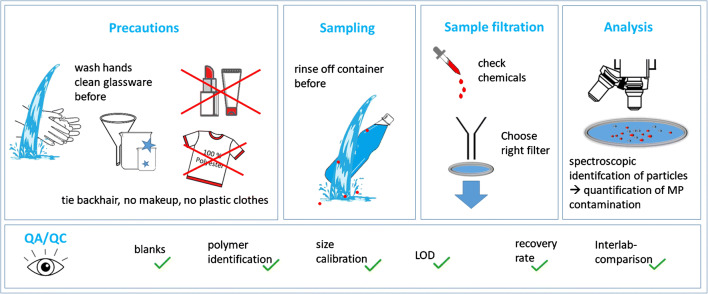

**Supplementary Information:**

The online version contains supplementary material available at 10.1007/s00216-021-03498-y.

## Introduction

Microplastics (MPs) are considered as a new class of contaminant whose emergence derives from the enormous growth in polymer use over the last 70 years and the progressive fragmentation of the resulting plastic waste debris dispersed into the environment [1, 2]. There is an ongoing discussion about the definition and categorization for the term “microplastics” [3, 4]. The Technical Report CEN ISO/TR 21960 defines “microplastic” as “any solid plastic particle insoluble in water with any dimension between 1 μm and 1,000 μm” and “large microplastic” as particles between 1 and 5 mm [5]. In contrast, the Californian State Water Resources Control Board currently defined “‘Microplastics in Drinking Water’ […] as solid polymeric materials to which chemical additives or other substances may have been added, which are particles which have at least three dimensions that are greater than 1 nm and less than 5,000 micrometers (μm). Polymers that are derived in nature that have not been chemically modified (other than by hydrolysis) are excluded [6].” Within this resolution, a size-based nomenclature differentiates between “nanoplastics” (1 nm to < 100 nm), “sub-micron plastics” (100 nm to < 1 μm), “small microplastics” (1 μm to < 100 μm), “large microplastics” (100 μm to < 5 mm), “mesoplastics” (5 mm to < 2.5 cm), and “macroplastics” (> 2.5 cm). Another distinction can be made between “primary” and “secondary” MPs. Primary MPs are intentionally manufactured plastic particles added to products for functional purposes, e.g., as abrasive material in toothpaste, exfoliants, and other cosmetics. Secondary MPs originate from larger plastic items which have degraded into smaller fragments (due to UV radiation as well as mechanical, physicochemical, and biotic factors), including MPs that are formed from plastic packaging during use such as abrasion from bottles and caps. These MPs may even break down into nanoplastics [7–11].

With increasing numbers of scientific publications on MP contamination in environmental matrices as well as in food and drinking water, the calls for harmonized methods become louder. In fact, this is one of the research gaps highlighted in the latest report of the World Health Organization (WHO): “a set of standard methods is needed for sampling and analyzing MPs in drinking-water and fresh water.” [12]. Moreover, due to the California Safe Drinking Water Act and the Directive (EU) 2020/2184 on the quality of water intended for human consumption, standard methodologies for monitoring MPs in drinking water are needed [13, 14]. To date, many different methods have been reported by numerous laboratories as summarized by Koelmans and colleagues who assessed the quality of MP detection in fifty studies analyzing water from rivers, lakes, groundwater, tap water, bottled drinking water, and wastewater [15]. From these studies, in which MP concentrations varied across ten orders of magnitude (10^−2^–10^8^ particles/m^3^), Koelmans et al. concluded that 92% of the reviewed studies were not fully reliable in certain quality assurance (QA) aspects [15]. Furthermore, a huge diversity between the experimental setups of the examined studies was shown. The review by Koelmans and colleagues emphasizes a lack of comparability of the results from different studies, which makes the monitoring and controlling of potential MP release nearly impossible. Instead, as Zarfl summarizes in her review, the “standardization of MP analytical methods on the basis of research aim will help to make study results comparable and obtain a more comprehensive picture of MP abundance and fate in the environment.” [16]. One important step toward reproducible and comparable studies was recently made by Cowger and colleagues, who reported a detailed guideline on a huge number of steps to be noted during microplastic analysis [17].

Apart from the absence of standardized sampling procedures [11, 12, 18, 19], the analytical difficulties in MP research are further compounded by the limited use or non-availability of common quality control (QC) measures [19, 20]. In particular, there has been a lack of analytical standards, appropriate reference materials, and interlaboratory comparison (ILC) studies. For the latter of these, the first initiatives were launched only recently [21–25]. The “Discussion Paper: Microplastics Analytics – Sampling, Preparation and Detection Methods” focuses on different physicochemical methods and analytical approaches in order to achieve valid methods and comparable results [3]. Recently, an updated version of the document “Status Report: Analysis of microplastics – Sampling, preparation and detection methods” was presented [26]. The first “Standardized protocol for monitoring microplastics in seawater” was published in 2019 as a result of the “BASEMAN” project [27]. It recommends procedures for MP sampling, processing, and analysis for surface and water column seawater samples, beginning with particles > 100 μm. Enders et al. published a protocol to extract MPs with sizes between 10 μm and 5 mm from environmental samples [28] and proposed several options to size-fractionate larger from smaller particles. Unfortunately, the proposed operating procedures are not applicable for the analysis of particles in the lower size range (especially below 10 μm).

The most common techniques to reliably identify MPs are spectroscopic (infrared (IR) spectroscopy and micro-Raman spectroscopy (or Raman microspectroscopy, RM)) or thermal degradation methods [19, 29, 30]. While the mass-based thermo-analytical methods can determine the overall mass of different polymer types, the particle-based spectroscopic methods provide information on the polymer type, number, size and size distribution, and morphology separately for all analyzed plastic particles in a sample [3, 11, 31].

Furthermore, in considering the applicability of the two approaches, it must be noted that drinking water samples can potentially contain high numbers of small (< 10 μm) MPs of several polymer types [32–34]. One spherical particle with a diameter of 10 μm and a density of 1 g/cm^3^ has a mass of 0.5 ng, while one particle with a diameter of 1 μm equals a mass of only 0.5 pg. Particles of such small size can still be identified individually via spectroscopic methods (RM). In contrast, thermo-analytical methods can only detect them, if they occur in large numbers so that their total mass (one polymer type) exceeds the limit of detection (LOD) of the method (which lies in the range of μg, [26]).

Other important types of information, especially in relation to toxicological concerns, is the number, size or size distribution, and shape of the MPs. It was, for example, shown that polystyrene (PS) particles below a size of 4 μm can pass human intestinal epithelium cells in minor fractions [35]. Spectroscopic methods are particularly well suited for gathering these characteristics, because they permit a direct enumeration of potential MPs and in contrast with colorimetric methods (use of dye) or morphological methods (e.g., scanning electron microscopy, SEM); they are capable of specifically determining their identity through a complete spectral pattern recognition. Given these advantages, this consensus paper focusses on spectroscopic methods as the means to satisfy the analytical requirements for MP analysis in clean water.

To this end, a group of 12 European analytical laboratories and institutions (from Germany, France, Italy, and Spain) experienced in spectroscopic MP analysis has discussed and developed a joint working paper, describing the consensus for sampling procedures and detection methods used in these laboratories. The present paper compiles all the information gathered in working meetings and discussions together with critical review of literature published by other authors and provides a guideline that describes minimum requirements and best practices for MP analysis by spectroscopic methods in clean waters. Clean waters, for the purpose of this consensus paper, comprise all waters with a low content of background matrix, in particular drinking water, bottled and tap waters but also other water types such as clean natural fresh waters or injectable water for medical/pharmaceutical applications, e.g., 1/ Ph. Eur. 2.9.19. [36]. While preparing the manuscript, an extensive literature research was conducted (until 31.03.2021) via the most common databases: ISI Web of Knowledge, Google Scholar, and SciFinder. The search terms included, among others, microplastic, analysis, identification, quantification, characterization, vibrational spectroscopy, Raman, infrared, and drinking water. Then the publications related to guidelines for Raman and IR analysis of microplastics were selected and discussed in this review manuscript.

In this paper, all relevant methodological steps toward a valid and reliable MP identification are described﻿ and discussed. It includes precautions and advice on sampling and sample preparation, avoidance of sample contamination, measurement methods, data processing, and method validation. In addition to their discussion in the text, all these points are summarized in Table 1. The minimum requirements and the best practice approaches are designed to (i) improve the reliability of MP analysis by helping labs to identify pitfalls in the analytical method and, thus, to avoid generating false-positive and false-negative results; (ii) facilitate and improve the planning of MP analysis; and (iii) provide a better understanding regarding the evaluation of already existing studies. Overall, the present review attempts to make an important step toward harmonization of MP analysis in clean waters in order to allow the comparison of results obtained in different studies by using similar or harmonized methods. These proposals and best practice approaches are intended to support the different standardization processes that are ongoing throughout national and international normalization committees.

**Table 1:** Minimum requirements and the best practice guidelines for the analysis of microplastics in drinking water and other clean water samples with micro-Raman and micro-infrared spectroscopy

Method	Minimum requirement	Best practice
Avoiding sample contamination		
Air purity, type of floor/wall	Clean lab, linoleum or tiling floor, no carpet	Controlled air flow, clean room
Type of extraction hood	Laboratory hood surfaces must be thoroughly cleaned with filtered liquid (e.g., water) to avoid microparticle contamination	Laminar flow cabinet
Type of lab coat, clothes, gloves	Cotton lab coat, beneath: avoid all clothes with potential release of synthetic textile fibers Gloves: if used, check critically for potential contamination	Hairnet, beard protector/guard
Operator precautions	Wash hands, tie back hair, if mask must be worn, use N95	No make-up, no hydration cream
Sampling		
Type of sampling container or online process	Clean containers Minimize plastic use during sampling	Glass or stainless steel—avoid plastic component/item
Volume of sample	Volume adapted considering the water type (number and size distribution of microparticles)	Volume adapted to the container (e.g., entire packed bottle) Avoid sub-sampling, if possible
Number of replicates	1	Minimum 3
Preparation of sampling container	Mechanical cleaning and rinsing (e.g., cleaning with particle-free water)	Filtrated tension-active/surfactant or chemical solution (e.g., sodium dodecyl sulfate, chromosulfuric acid)
Sample preparation		
Cleaning of the outer side of the sampling container	Mechanical cleaning and rinsing before entering the lab hood/clean bench	
Addition of reagent, use of tools	If adding reagents: check for purity/possible contamination through blank samples; filtration advised with further check of contamination Use of pre-cleaned glassware and tools (glass pipette) Avoid plastic tools/pipettes	Option to dissolve minerals: Na_4_EDTA solution (250 g/l) depending on the amount of calcium and magnesium in the sample prepared from Na_4_EDTA > 99%, filtered through a syringe membrane filter (e.g., cellulose acetate, 0.2 μm)
Sample filtration		
Filtration steps	1	Optional: fractionated filtration, for example, with multiple filters of different pore sizes (same material or any other approved material)
Nature of filter	Free choice	Check the quality of the filter surface: it should be default-free and very flat For automated systems: • Silicon (FTIR, Raman) • Metal (Au, Al)-coated PC filter (IR, Raman): exclusion of PC results advised; at least careful checking of the level and stability of the blank needed • Aluminum oxide (IR) • PTFE (Raman): exclusion of PTFE results advised; at least careful checking of the level and stability of the blank needed For non-automated systems: Free choice, e.g., above-named filters or others, e.g., nitrocellulose
Filter features (pore size, dimensions)	Filter pore size must be adapted to the size of particles delivered in the report (information about filter pore size should be given in the report)	To reduce the time of analysis, the smallest possible filter surface should be chosen If background correction is applied in spectroscopy, pay attention to the distances between pores to obtain adequate background signals from the filter
Nature of filter holder and other materials used	Avoid plastic tools as alternatives are existing Use of plastic devices (e.g., PTFE) needs critical and very strict control (check blanks) with possible exclusion of corresponding polymer particles	Stainless steel, glass, or colored PTFE (colored with blue or red dye; allows the laboratory to give results for PTFE, with exclusion of the dyed material from the results)
Volume of sample filtered	Sub-filtration possible; if it is done, it has to be stated in the report	Ideally filtration of the entire sampling volume to reduce inhomogeneity when aliquoting the sample
Rinsing conditions after filtration		Glassware rinsed once with particle-free water after initial filtration of the sample to maximize the recuperation of microparticles potentially stuck at the surface of glassware. Be aware that the rinsing step might bring contamination (check blank values)
Handling, transport, and storage of the filter	Protection of the filter from atmospheric deposit needed, e.g., in glass Petri dishes or metal containers It is highly recommended to avoid plastic containers (possible contamination; electrostatic charging may result in a loss of particles)	
Laboratory blanks		
Matrix used	Particle-free water: tested in the lab or water bought and tested	
Frequency of blank samples during routine analysis	1 blank per series or day for a maximum of 10 samples	More than one blank per day or 10 samples
Acceptance criterion for blank routine analysis (MPs/blank), for validation or invalidation of the series	The sum of all kinds of MPs in the blank sample must not exceed the LOD of the method (see below) for accepting the results of the series If the number of MPs in the blank sample is higher, some contamination occurred during sample processing that could have polluted the samples, leading to false-positive results *Exceptional* procedure: If a high contamination with only one polymer type occurs, the lab can exclude the results of this polymer and give additional information about the contamination (number, size, etc.)	
Analysis		
Particle detection mode	Parameters for image acquisition (e.g., focus, contrast/brightness) have to be adapted in order to obtain correct images for particle detection (e.g., for correct size determination)	Auto or semiautomatic particle detection possible. Dark-field illumination can be used to improve the detection of small particles (< 5 μm). Critical parameters for particle detection may ideally be adjusted automatically or should be fixed in order to avoid inter-operator bias. Less important for mapping/imaging during Raman/IR measurement.
Size range of MPs targeted (μm)	Information on smallest particle size analyzed (size range and distribution) If particle numbers are reported in a binned form, the following size classes should be used: 1–5 μm, 5–10 μm, 10–20 μm, 20–50 μm, 50–100 μm, 100–500 μm, > 500 μm	For future best practice, the specific particle sizes, e.g., in the form of raw data, should be provided for further data analysis and modeling.
Libraries used	Minimal included polymer types: PE, PET, PP, PS, PC, PVC, PMMA, PTFE, nylon (PA), PU (several types) Natural materials present in samples (e.g., proteins, cellulose) to avoid mistaking with, e.g., PA (see Supplementary information (ESM), section S1)	Additionally, spectral data for additives (e.g., pigments (Raman)), elastomers, further naturally occurring materials (e.g., minerals) Homemade spectral database, for example, including materials from sample packaging, containers, and materials used in the laboratory
Match acceptance criterion between sample spectra and database reference	If the laboratory is using a fixed limit for automatic acceptance of spectral matching with the database (e.g., hit quality index, HQI): The minimum limit can be set at a matching result of, for example, > 70% The lab has to approve initially that the automatic identification for spectral matching above this value is correct, e.g., through operator/human review of the characteristic peaks in the spectra. Afterwards, the lab is free to consider particles with spectral matching below this value as identified, when the identity is confirmed via operator/human review. Be aware that different software may produce different values for the HQI and that a verification of the algorithm has to be done to confirm the correct identification of the material (see “Data processing”). Besides classical database search, other identification techniques, such as a homemade semiautomatic identification via mathematical algorithms (e.g., classical least squares (CLS), including manual review of results), model-based classification (e.g., random decision forest (RDF) classifiers) are possible after validation of the recognition model.	Pay attention to the spectra of nylon and proteins, which are very similar in IR and Raman spectra (compare “Data processing” and ESM, section S1). The same kind of spectral similarities could issue with Polyethylene and molecules containing long CH-chains, e.g., stearates leading to potential false-positive identification.
Objective used	Depends on samples and equipment type—must allow to obtain a good image/signal	Adjust the objective to the analyzed particle size, e.g., for Raman, particles of 1 μm can be measured with a 50× objective
IR acquisition mode		Transmission and reflection modes for micro-(FT)IR are easier/faster to use ATR: only for particles > 100 μm. Slower, more difficult to use and attention must be paid to the cleaning of the device (germanium/diamond) with contact mode to get rid of any cross contamination
Range of acquisition (cm^−1^)	IR: 1250–3600 cm^-1^ Raman: 200–2000 cm^-1^	IR: entire MID-IR range Raman: 50–4000 cm^−1^ (Raman)
Raman laser wavelength used (nm)	532 nm or 785 nm	
Raman laser beam spot size (μm)	Spot size depends on the instrumentation (information on the spot size has to be given in the report)	Down to 1 μm, if particles of that size have to be analyzed
Raman laser parameters	Laser parameters should not cause particle destruction (RM) Acquisition time minimal 1 s for single particle measurement to reach an acceptable signal-to-noise ratio. For imaging, shorter time can be used.	Measurement time as long as necessary to get good spectra, but as short as possible to save time. ADVICE: Work as much as possible in non-destructive mode or with low laser power. Some examples are given for a mean generic value to start the acquisition testing of a sample: silicon filter/magnification 20× (NA 0.50) or 50× (NA 0.55)/laser power (532 nm) 5 mW or 6 mW, (785 nm) 15 mW Au-coated PC filter/magnification 20× (NA 0.50) or 50× (NA 0.55)/ laser power (532 nm) 3 mW/5 s, 10 s or 20 s Al-coated PC filter/magnification 50× (NA 0.55)/laser power (532 nm) 3 mW–(785 nm) 5 mW/2 s
Spectral resolution (cm^−1^)	IR : ≤ 8 cm^-1^	
Number of particles/surface of filter analyzed	Different approaches are possible to analyze particles on the filter. Different possibilities are listed below (beginning with the most favorable model) **(1) If an automatic counting system/imaging for particles is used**, the *whole surface* of the filter should be scanned for the total number of particles. Depending of this number, the following models can be used: (1 A) THE TOTAL SURFACE MODEL If the total number of particles is < 1000 (< 500 for practical reasons, < 5000 or 7000 for best practice), all particles on the filter surface should be analyzed by spectral recognition. If the total number of particles is > 1000: ALL particles > 50 μm (up to 1000) have to be counted and measured (if possible with imaging/sizing system), especially for environmental or complex (e.g., food) samples. For particles < 50 μm, one of the following models should be chosen: (1 B) THE RANDOM MODEL Choose randomly a selected number (1000 particles at minimum, 500 for practical reasons, 5000 or 7000 particles for best practice) to be analyzed/identified. (1 C) THE “CAKE” MODEL If random particle selection is not possible, at least one region representing “a piece of cake” (from the center of the filter to the border of the filter, for example, a quarter of the filter is a piece of cake by 1/4) has to be chosen for analysis. Its surface should be at least 20% of the total filter surface, when analyzing particles down to 10 μm (IR) or 5 μm (RM), and at least 4% of the total filter surface, when analyzing particles down to 1 μm. Additionally, the number of particles analyzed on this piece of cake must exceed 1000 (500 for practical reasons, 5000 or 7000 particles for best practice). (1 D) THE HELIX or “SNAIL” MODEL If it is technically not possible to choose random model, at least 5 regions on the filter have to be chosen for analysis. Their total surface should be at least 20% of the total filter surface, when analyzing particles down to 10 μm (IR) or 5 μm (RM), and at least 4% of the total filter surface, when analyzing particles down to 1 μm. Additionally, the number of particles analyzed for the chosen regions must exceed 1000 (500 for practical reasons, 5000 or 7000 particles for best practice). **(2) If there is no automatic counting system/imaging for particles** - All particles on the entire filter surface must be analyzed with spectral recognition up to a total number of particles of 1000 (500 for practical reasons) as minimum requirement (> 2000 for best practice approach) - If the total number of particles is > 1000, use a combination of: Analysis of all particles > 50 μm up to 1000 (500 for practical reasons) (possible with imaging/sizing system) AND selection of one statistical model from B to D for particles < 50 μm. The final results are obtained as the sum of the particles above and below 50 μm (measured with one of the above-described models; each extrapolated to all particles detected in the corresponding size range).	Spectral recognition of all particles on the filter, if possible. If too many particles occur on the filter, a *representative* aliquot can be taken to decrease the total number of particles (see “Models for sub-sampling of particles deposited on a filter”).
Method validation		
Description of the way to validate the method	I. Verification of size measurement of the equipment, e.g., with particles of known size II. Verification of qualitative polymer identification at the claimed minimal size, at least for the main polymer types (PE, PET, PC, PP, PVC, PS, etc.) III. Determination of the LOD as the mean of all MPs identified in 10 blank samples + their threefold standard deviation: *LOD* = *mean*_10 *blanks*_ + 3 ∗ *s*_10 *blanks*_ Re-determination after modifications of the method	IV. Verification of the recovery rates of the entire method V. Inter laboratory comparison (ILC) and proficiency tests (PT)
Data processing		
Information to be given in the report	• Total number of particles in the sample or sub-sample (if available) • Number of particles analyzed • LOD • Number of total microplastics identified (calculation or measurement) ➢ By type of polymer ➢ By size ranges ➢ No blank subtraction • If sub-sampling during measurement was done: analyzed area of the filter (%) or statistical percentage of analyzed particles on the total number of particles (%) The laboratory should report quantitative results only, if the results exceed the LOD. Otherwise, results can be given as < LOD.	Additional information, if requested: • Shape (fragment, fiber, or bead) • Color, pictures of particles or filters • Number of (potential) MPs not included in the minimum set of polymer classes. Total number of non-plastic particles identified (e.g., minerals, proteins, cellulose), by type, by size range, etc. • Total number of non-identified particles If qualitative information is given about particles smaller than the pore size of the filter, it must be demonstrated that these particles were intrinsic to the sample. Further, it must be stated that these data are not representative. For future best practice, the specific particles size, e.g., in the form of raw data, should be provided for further data analysis and modeling.

## Measures to avoid sample contamination

**Fig. 1:**
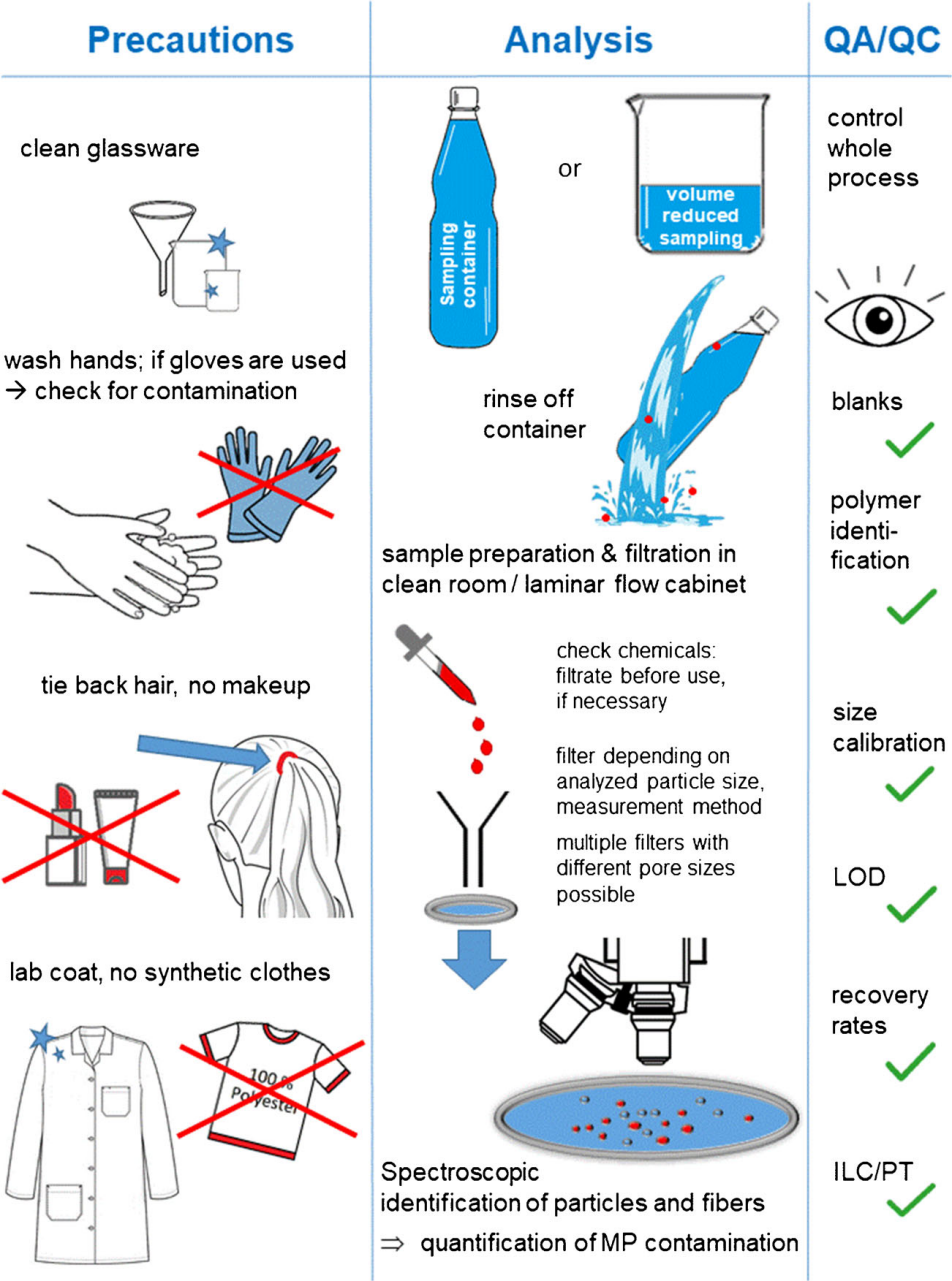
Important precautions and advice for the analysis of microplastics

The laboratory working environment is a critical point for analysis of MPs. Koelmans and colleagues [15] pointed out that laboratory working conditions represent a key factor in improving the quality and comparability of results. Some recommendations have already been described to improve the quality of analytical methods [3, 12, 20, 37]. These include the avoidance of plastic items and synthetic components during the entire analytical process from sampling to analysis. Figure 1 gives an overview of critical points concerning a quality-controlled microplastic analysis.

As a starting point, the laboratory should be largely free of MP particles or fibers. In order to ensure this, the floor of the laboratory needs to be of an easily washable material and must be regularly cleaned. Some laboratories use sticky anti-contamination mats at the entrance [20]. Airborne particles and fibers are one of the major causes of external sample contamination [38] and must be assessed and minimized by appropriate strategies. As described in previous studies, the use of controlled air flow is recommended to maximize air purity and minimize airborne contamination [15, 33]. Sample handling should be performed in a “clean air laboratory” [39–41] or in a laminar flow cabinet [3, 42]. By choosing a laminar flow cabinet, at least ISO Class 5 (3520 particles/m^3^ for particles ≥ 0.5 μm) is advisable, whereas a cabinet of ISO Class 3 (35 particles/m^3^ for particles ≥ 0.5 μm) [43] is preferable, especially for the analysis of MPs in the lower μ-range. Additionally, surfaces in the lab and the laminar flow cabinet must be cleaned regularly [20].

Due to the risk of contamination by plastic microfibers, clothing requires special attention [37]. In all handling steps, cotton or antistatic lab coats (particle free) must be worn by all analytical staff. Moreover, clothes containing synthetic fibers, even when worn underneath a lab coat, may increase the ambient level of MPs [19]. General precautions during sample handling have to be followed by operators in order to decrease the risk of contamination (e.g., wash hands, tie back long hair, no make-up, nail polish nor hydration cream), as these products may contain and release MPs [44].

Wherever safety considerations permit, the use of disposable laboratory gloves should be avoided, as they might cause sample contamination. If necessary, the usage of gloves must be critically checked by analyzing blank samples [45]. Moreover, recent pandemic events may impose the need for laboratory staff to use temporarily specific personal protection equipment as (single use) masks. These masks can be made from polypropylene (PP) and thus might release numerous PP microparticles into the lab environment [46]. In general, labs are encouraged to critically reflect on any temporary modification of usual working practices that could interfere with MP measurements.

Cleanliness of the working area needs to be evaluated by the operators. Schymanski et al. performed a periodical evaluation of particle levels with a particulate measuring device inside the hoods to verify their efficiency [33]. Some authors deposit empty filters or petri dishes next to the working area to check for background contamination levels [47, 48].

To evaluate the contamination level in parallel with every sample batch, a blank test with particle-free water (recognized quality, e.g., ultrapure, ultra-filtrated, or pyrogenic water) should be performed (see “Laboratory blanks”).

Plastics may only be used if the corresponding polymer is excluded from the results or if it is marked, for example, with a fluorescent dye and can thus be distinguished from sampled MPs. For instance, some laboratories use filtration devices that contain parts made from specifically colored polytetrafluoroethylene (PTFE), which enable the recognition of other PTFE particles that may originate from the sample [49]. If possible, only glass or stainless steel should come into direct contact with the sample. In addition, it is essential that containers for sampling are thoroughly cleaned prior to use [15]. This can be achieved by mechanical cleaning and rinsing with particle-free water. In order to remove particles firmly stuck to container walls, this process may not suffice. Further options for particle removal are baking of containers in a muffle oven [50], sonication of containers filled with water [34], and rinsing with pre-filtered surfactants or chemical solutions. Potential sample contamination during sampling should be monitored by the analysis of blank samples (see “Laboratory blanks”) [15, 20].

Experiments showed that 20 to 80 MPs down to a size of 10 μm are present on the external surface of a bottle/container and could be removed by rinsing the outer surface in order to preserve the bench and the hands of manipulator from this contamination (unpublished data by N. Benismail). Therefore, it is necessary to clean the outside of the container, e.g., with particle-free water and a detergent. Furthermore, within this step, labels should be removed from bottled water whenever possible. A final rinsing with particle-free water should be performed before placing the sample containers in the laminar flow box, where the sample containers are left to dry prior to further handling [32, 33].

In order to avoid sample contamination by adding chemicals, it is highly recommended that any particles present in the reagent solutions are removed by pre-filtering the chemicals [20, 51]. This can, e.g., be done with syringe membrane filters (with 0.2 μm pore size, e.g., made of cellulose acetate [32]). Instead of syringe membrane filters, the chemicals can be filtered through a polycarbonate (PC) filter (with a pore size of 1.2 μm) as proposed by Weber et al. [52]. Alternatively, 1.2 μm and 0.7 μm glass-fiber filters can be applied for the filtration of the reagents before use, as reported by Johnson et al. and Kirstein et al. [53, 54], respectively. But, it has to be pointed out that the pre-filtering of chemicals can lead to a contamination with MPs, e.g., particles can be released from the filter membrane, the plastic housing of the membrane filters [55] or from frits of classical filtration devices (unpublished data of B. E. Oßmann). Therefore, both filtered and non-filtered chemicals should be tested for particles in order to decide on a case-by-case basis if filtering is suitable or not.

After filtration, during the transfer to the analytical device and for storage, the filter has to be covered to avoid contamination from the air. Different container types can be used, such as glass petri dishes or metal containers. It is highly recommended that the use of plastic materials (e.g., polystyrene (PS)/PC petri dishes) is avoided.

## Sampling

In the case of MP analysis, samples typically contain a heterogeneous mixture of particles, of which only a very small percentage is actually MPs. Therefore, it is extremely important to analyze this heterogeneity and apply appropriate sampling, as well as sample preparation tools and methods, which shift the system toward a state that guarantees a representative analysis of the lot [56]. Because particles in the size range of 1 μm to 1 mm have a very small mass, it is advisable not to prematurely focus on sample mass, but rather on particle numbers.

Sampling strategies for microplastic analytics in water vary a lot between different water types. Bottled waters are usually sampled as entire bottles/packages and brought to the lab as they are [32, 33, 57]. In contrast, for tap waters, a defined volume is taken with sampling containers (e.g., glass bottles [34, 58]) or the volume is reduced on-site with special filter cartridges [53, 54, 59, 60]. In order to provide representative results, it is important to adapt the sampling volume to the anticipated properties of the sample [20]. In waters, which are expected to have low MP concentrations (e.g., tap water), a much higher sampling volume is required in order to produce representative results, than in waters with higher anticipated MP concentration [15]. The number of (plastic) particles in the sample increases by orders of magnitude with decreasing particle size. Therefore, usually a lower sample volume is required for representative sampling of particles in the smaller micrometer size range. Different research groups, who analyzed MPs > 25 μm, > 20 μm, > 6.6 μm resp. and > 5 μm in tap waters sampled several hundred to several thousand liters [53, 54, 59, 60], while Pivokonsky et al., who analyzed MPs >1 μm in tap water sampled only one liter [34]. If achievable, ideally a triplicate of each sample should be taken and analyzed (best practice) [20].

## Sample preparation and filtration

Particles are usually separated from the water matrix via filtration, either on-site during sampling (see “Sampling”) or in the laboratory. In the first case, the filter residue is transferred from the cartridge to another filter in the laboratory; in the second case, samples are filtered through a filter, which is suitable for analysis. The applicability of a filter material depends on the analytical technique that will be used and the particle size to be analyzed.

In general, filters suitable for analysis have to be flawless and flat in order to gather all particles of a similar size within one focus [61]. Filter flatness can be enhanced by using special membrane-flattening holders during measurement [62–64].

The pore size has to be smaller than the minimal particle size, which is reported quantitatively. However, pore size may not be chosen far below the targeted particle size, as a larger pore size simplifies and speeds up filtration and prevents the filter from being clogged or covered with matrix residues [51, 65]. What is more, the filter material must allow optical particle recognition (for visual analysis and methods based on image analysis) and must not interfere during measurement [61, 66].

For RM, either silicon membranes or metal-coated (Au or Al) PC filters are recommended. Both filter types obtain high contrast to particles, if dark-field illumination is applied [33, 61, 66]. Besides, PTFE membranes are suitable, when used in bright-field illumination [60]. If particles are selected and analyzed manually, other filter materials like nitrocellulose are also adequate [67, 68]. Gold- and aluminum-coated PC membranes do not interfere with particle spectra as they do not show an own intense Raman spectrum [33, 61]. In contrast, other filters, such as PTFE, silicon, or cellulose, show characteristic peaks themselves. While the spectra of PTFE and silicon can easily be distinguished from targeted plastics, cellulosic filters can interfere during MP identification [60, 61, 66].

Using IR spectroscopies, one has to differentiate between reflection and transmission measurements. For reflection measurements, gold-coated PC membranes have been applied successfully [69, 70], as well as reflective microscope slides [71]. Further, other light-reflecting surfaces like filters and microscope slides coated with silver or other metals are conceivable [30, 70]. For transmission measurements, substrates must be transparent for IR light in the relevant spectral range. Aluminum oxide membranes (Anodisc) [72], zinc selenide windows [73], or silicon filters [66] are commonly used materials. Anodisc membranes are only infrared transparent between 3600 and 1250 cm^−1^, which partially masks the IR fingerprint region (1400–600 cm^−1^). Nevertheless, most polymer types can be identified [63, 72, 74, 75]. In contrast, silicon filters, which are transparent for the broad mid-infrared range (4000–600 cm^−1^), allow measurement of the IR fingerprint region, while being available in a variety of pore sizes in the micron range. This facilitates sample preparation, while providing a very flat surface. However, custom-made filtration units are necessary as these membranes are typically square-shaped [66].

Some filter materials consist (partly) of plastics (e.g., PTFE, metal-coated PC membranes, and Anodisc with PP support ring), which might lead to sample contamination. When using such filters, it is necessary to critically check this potential contamination route via the analysis of blank samples (see “Laboratory blanks”). If in doubt, it is advisable to exclude this polymer type from MP results to avoid misinterpretation [33].

The sample volume (see “Sampling”) filtered for the actual analysis must allow representative analysis and at the same time prevent overloading or clogging of the filter. In order to achieve these goals, one can use different options. Reduction or sub-sampling of the initial sampling volume should be avoided whenever possible, as this will introduce a larger error margin. If this is not possible, information about sub-filtration has to be reported. E.g., for bottled water, it is advisable to filter the entire bottle volume, but when analyzing MPs down to 1 μm, the filtration volume has to be reduced to get a measurable particle density on the filter [32].

Besides sub-sampling or reduction of the filtration volume, it is possible to use fractionated filtration with filters of different pore sizes to reduce particle numbers and avoid filter clogging. Subsequently, all the filters have to be analyzed separately for MP of different size categories [34, 49, 60]. If samples contain a high amount of background matrix, it may be necessary to add chemicals to the samples prior to filtration or to treat the residue of volume-reduced sampling before transfer to the filters for analysis. Alternatively, one might treat the residues deposited on the filter with chemicals after filtration. For example, Mintenig et al. added hydrochloric acid to dissolve calcium carbonate and iron precipitates (e.g., iron oxides) after filtration [59], whereas Pivokonsky et al. used wet peroxide oxidation to digest organic material in tap water samples [34]. Another option to dissolve carbonate particles is via complexing calcium and magnesium cations by adding an equimolar amount of ethylene diamine tetraacetic acid tetrasodium salt as solution [32]. In order to reduce the risk of sample contamination by the addition of chemicals, such sample treatment steps should only be done if necessary.

The actual filtration area should be reduced to a minimum to decrease measurement time. At the same time, one has to ensure that particles do not overlay, inhibiting correct particle analysis. For example, Schymanski et al. applied a filtration area of 12.6 mm^2^ (pore size 3 μm) to filter up to 1.5 l of mineral water, whereas Oßmann et al. applied a filtration area of ~ 113 mm^2^ (pore size 0.45 μm) to filter 250 ml of mineral water [32, 33]. Before filtering the sample, homogenization should be achieved, e.g., by gentle shaking. After filtration, the sample container and the funnel of the filtration device can be rinsed with particle-free water or solvents to recover particles adhering on the walls.

## Laboratory blanks

Careful attention is required to ensure that samples are not contaminated by external particles [15, 33, 38]. For QC and in order to quantify potential external contamination, reagents and materials used for performing sample analysis (e.g., water and chemicals used for rinsing and cleaning of utensils and containers), as well as the entire procedure from sampling to analysis must be controlled [20].

Therefore, particle-free water must be analyzed regularly as a process blank. This blank sample must undergo the same procedure as the actual samples, including all sample preparation steps [76]. For a small series with less than ten samples that are all processed within one day, a single (process) blank sample is sufficient. For large series with more than ten samples, multiple (process) blank samples should be analyzed alongside the sample series (e.g., at least one process blank sample per five or ten samples) [26].

The number of MPs (as a sum of polymers) found in the process blank sample indicates the level of external contamination that has reached the samples during the daily sample manipulation. To validate a series of samples, the particle count from the blank should be compared to the values obtained during the validation stage of the method (see “Method validation and quality controls”). If the result from the daily process blank sample is lower than or equal to the LOD determined during the initial validation of the method, the daily sample manipulation can be considered to have been correctly done in agreement with the given recommendations of this paper. On the contrary, if the number of MPs in the process blank sample is higher than the LOD, the series of samples has to be invalidated. Even when all recommended precautions are taken, unexpected external contamination may still occur in the process blank sample and thus may also have reached the samples.

In the case where the number of MPs detected in the blank value is higher than the LOD, but this value is attributable to a high number of MPs of one polymer type only (with the sum of the others being below the LOD), the results for all other polymer types of the sample series may be considered as valid. The conspicuous polymer type must be excluded from all results for this sample series. This procedure should only be used as an exception. Furthermore, as much information as possible should be provided on the contamination (e.g., particle number, size, conspicuous polymer type, potential contamination source).

To insure that the lab is reporting correct values, it is strongly recommended that sample results are presented without prior subtraction of blank values. The LOD of the lab should be expressed each time results are given. When sample results are below the LOD (see “Method validation and quality controls” and “Ways of reporting results and valuable information”), these results should be expressed in the report as < LOD.

## Analysis

### (Fourier transform) infrared (FT)IR spectroscopy

The key parameters that can be adjusted for each (FT)IR instrument are the spectral range; the spectral resolution, i.e., the ability to resolve features within the chosen spectral range; and the number of accumulated sample spectra. Optimizing these parameters on any given instrument is crucial to obtain high-quality spectra [72]. In many cases, a spectral range from 3800 to 900 cm^−1^ at a resolution of 8 cm^−1^ with 6 to 30 sample scans is applied [64, 72, 73].

Different approaches can be followed to identify particles by (FT)IR spectroscopy: Clean individual particles can be measured using an attenuated total reflection (ATR) accessory. ATR-FTIR spectroscopy is limited to relatively big particles (approx. > 100 μm) that can be handled individually.

The most common method of applying FTIR spectroscopy to MPs is micro-FTIR (μ-FTIR) spectroscopy, where a FTIR spectrometer is coupled to an optical microscope. Because of the diffraction limit, FTIR microscopy’s spatial resolution is physically limited to approximately 10 μm [64, 70, 74, 77]. This limit is further influenced by the microscope objective’s numerical aperture according to the Rayleigh criterion. μ-FTIR spectroscopy can be performed in reflectance or transmission mode. The choice of mode depends on the substrate (e.g., filter) onto which the MPs have been deposited. Alternatively, the substrate needs to be chosen depending on the method desired or available (see “Sample preparation and filtration”).

Reflectance mode, on one hand, is prone to undesired light-scattering effects on the particle surfaces that lower spectral quality [30, 78]. Light-scattering effects also interfere when analyzing small particles in the so-called transflectance mode. The IR light beam fully transmits the particles being then reflected from the filter surface, resulting in transmission-like spectra [77]. On the other hand, in reflection measurements, when the IR beam is focused on the particle surface, the radiation only penetrates a few micrometers deep into the particle (wavelength-dependent) [69]. This allows an investigation of the particle surface for polymer modifications due to aging effects or influences of sample preparation processes. In addition, reflectance FTIR spectroscopy has been successfully applied for the comparative FTIR and Raman spectroscopic analysis of MPs [70]. Transmission measurements were shown to yield high-quality spectra; however, thick (polymer-dependent, about > 100 μm) or colored particles may lead to total absorption in transmission mode. Consequently, IR bands converge resulting in unidentifiable spectra. Therefore, the most suitable IR technique should be chosen carefully.

Regardless of whether reflection or transmission measurement is used, a background spectrum needs to be recorded. This is necessary to account for noise from IR active substances other than the target MPs such as water vapor, carbon dioxide in the ambient air, or the substrate the sample is deposited on. Silicon membranes, for instance, show weak bands in the mid-infrared range, like the Si–O–Si stretching vibrations at 1108 cm^−1^ [66]. This background noise can significantly hamper sample analysis, but can easily be corrected. Before each sample measurement, a background spectrum is recorded similar to a sample spectrum on an unused filter or a clean spot on the sample filter or respective substrate. All measurement parameters must be the same as for samples. Furthermore, the number of co-added background scans must be at least as high as the number of sample scans [54, 79]. The instrument software usually automatically corrects the sample spectrum through subtraction or division of the background spectrum [77, 80].

μ-FTIR analysis can be very time-consuming, when measuring hundreds or thousands of particles one by one. Operator input time can be reduced with automated particle detection and FTIR measurement by using appropriate commercial or open-source software (e.g., GEPARD; see “Raman microspectroscopy”).

Measurement time can be accelerated significantly by coupling a focal plane array (FPA) detector to the FTIR microscope. These detectors comprise up to 256 × 256 MCT (mercury cadmium telluride) detector elements. Consequently, FPA detectors allow the simultaneous and therefore rapid acquisition of thousands of IR spectra in parallel. Furthermore, these spectra are spatially resolved, resulting in so-called IR images of well-defined sample areas. In this way, IR images of entire sample filters can be recorded in relatively short times compared to single-point detectors. Thus, for particle-rich samples, this area-based FTIR imaging can be quicker than a particle-by-particle approach and can be performed automatically, using, e.g., open-source software siMPle [79]. Automated FTIR imaging avoids the need for sub-sampling of the filter surface, but produces huge amounts of data that need to be analyzed. Furthermore, flatness of substrates is even more important (see above).

A very seldom used, yet noteworthy, technique is μ-ATR or ATR imaging, which combines μ-FTIR spectroscopy, ATR microscope objectives, and FPA detectors. ATR spectra are, in comparison with transmission and reflectance spectra, less prone to noise, light-scattering effects, and total absorption. However, this technique requires the sample to be deposited on a firm surface that does not break when the ATR crystal is pressed on it [63]. Moreover, particles tend to stick on the crystal, which makes any remeasurement of the sample area impossible.

A new technique, quantum cascade laser-based hyperspectral IR spectroscopy (QCL-IR microscopy), has recently been brought to market. This technique is also called laser direct infrared imaging (LDIR). Unlike traditional FTIR imaging systems, QCL-IR imaging systems make a first scan of the whole sample at one particular wavenumber to identify potential regions of interest. The regions of interest are subsequently scanned over a wider wavenumber range to gather enough spectral information to be able to identify the particles. For instance, Primpke et al. scanned environmental samples at 1470 cm^−1^ to identify potential MP particles [81]. These were subsequently scanned in the region 1800–1184 cm^−1^ and 1160–1084 cm^−1^ to identify the targeted polymer types. They compared the QCL-IR system’s performance to state-of-the art FTIR imaging and concluded that the results gained using QCL-IR microscopy were in good agreement with those of the reference method, while being about tenfold as fast [81]. QCL-IR, until now, was successfully applied to samples of soil [82, 83], river [84], and brackish waters [85]. However, further research is needed in order to clarify the feasibility and limitations of this technique for the MP analysis in clean water samples.

### Raman microspectroscopy

Raman microspectroscopy (RM) is a non-destructive analytical method based on the effect of inelastic light scattering on molecules. RM (analogous to IR spectroscopy) provides vibrational fingerprint spectra. The coupling of Raman spectroscopy with confocal optical microscopy and the use of a laser in the visible range allow a significantly better spatial resolution down to 1 μm and even below (down to approx. 300 nm) compared to μ-(FT)IR spectroscopy [31, 86, 87]. RM offers the advantage of being insensitive to water, which allows the analysis of MP in aqueous and wet samples. A major disadvantage of RM for the analysis of MP in real samples is interference due to fluorescence, which can be caused by inorganic (like clay minerals or dust particles), organic (like humic substances) and (micro)biological impurities in the matrix. Therefore, the choice of suitable measurement parameters (laser wavelength and power, photobleaching, and acquisition time, as well as objective magnification and confocal mode) is important to minimize or avoid interference from strong fluorescence and to improve the quality of spectra. In addition, removal of the (in)organic matrix from complex samples (e.g., [28]) leads to a significant increase of the plastic to non-plastic particle ratio. The spectral range for the Raman analysis of MPs has to cover all spectral features of (synthetic) polymers from 50 to 4000 cm^−1^ (best practice, or from 200 to 2000 cm^-1^ as minimum requirement). For the proper identification, both the fingerprint area (50 cm–1,500 cm^-1^) and the area including C-H stretching modes of alkyls, alkenes, and aromatic compounds (2800 cm^-1^–3200 cm^-1^) [87] are important. The entire measured spectral region has to be also covered by the databases, applied for the assignment of MP spectra. Thus, a reliable detection, identification, and quantification of MPs can be achieved. Additionally, not only synthetic polymers, but also additives (e.g., pigments), can be identified by means of RM. Furthermore, complementary information about the sample can be obtained by combining RM with μ-FTIR analysis (e.g., identification of pigment(s) and acrylic resin in paint particles [86]).

Raman imaging is usually performed as point-by-point measurements and is thus time-consuming [86], especially when particles smaller than 10 μm are to be analyzed. In contrast to an FPA-FTIR approach, Raman imaging is likely to be less effective than particle-by-particle measurement [62].

Since a very high number of particles have to be measured in order to achieve representative results (see “Models for sub-sampling of particles deposited on a filter”), automation of the RM is necessary. Currently, both commercial and open-source (GEPARD [49] and *TUM-ParticleTyper* [62]) programs can be used for automated RM analysis. This way, the analysis of the initial optical image provides morphological characteristics and coordinates for all analyzed fragments, while subsequent Raman measurement delivers their chemical identity. From practical experience, the following parameters/options have proven to be well suited for automated measurements: 532 nm (in some cases also 785 nm, e.g., fluorescent samples) laser excitation wavelengths, dark-field (in some cases also bright field) illumination, and objectives with longer working distance.

Before spectrum acquisition, Raman systems must be calibrated. Depending on the instrument, this is commonly done on the 520.7 cm^−1^ peak of a silicon wafer and by zero-order correction of the used grating [88, 89]. Furthermore, some Raman instruments allow for an automated calibration in the broad spectral range using an internal Ar/Hg calibration lamp [90].

### Models for sub-sampling of particles deposited on a filter

There are two ways of sub-sampling: the samples may be split before filtration (see “Sample preparation and prescription”), and subsequently, the whole filter is analyzed or alternatively, the whole sample may be filtered and then only a fraction of the filter surface is analyzed.

**Fig. 2:**
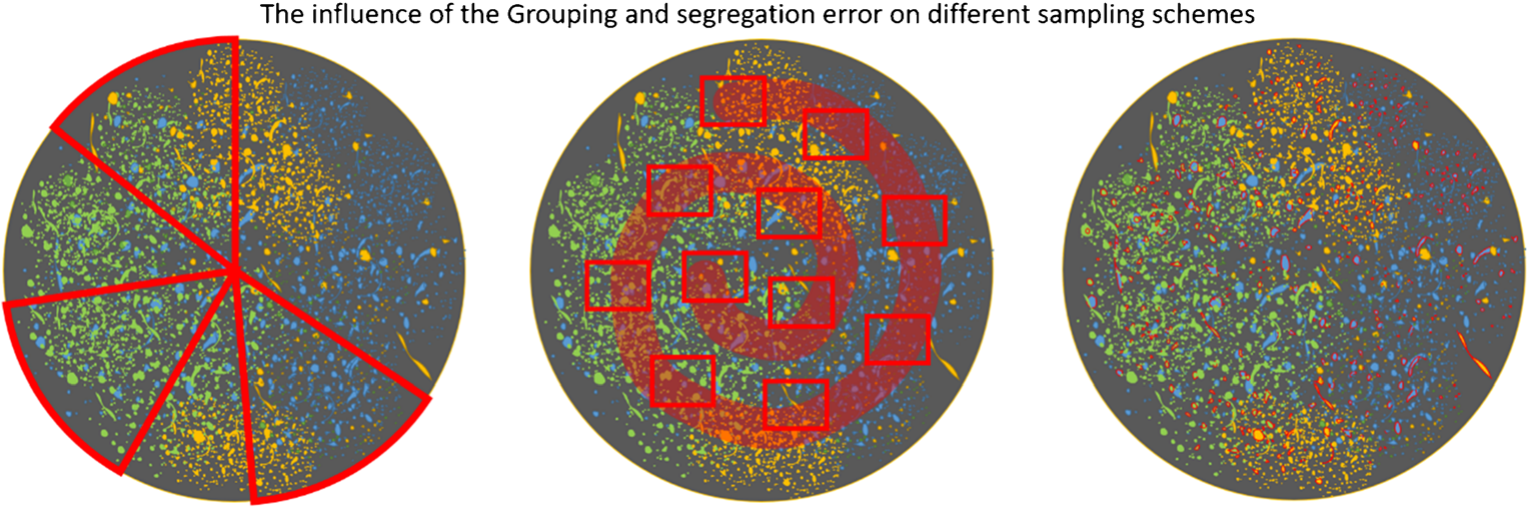
Illustration of the three sub-sampling models and their effects on introduced errors. Left, “cake” model. Slices are selected for measurement. Depending on their location and size, the grouping of particles may affect the results and introduce errors. Middle, “snail” model. A composite sampling strategy based on the selection of multiple small boxes, which should be spread across the filter, so that the edges and middle are represented. Right, random model. Since each fragment is randomly selected, the grouping of particles does not influence their selection. Representativity is only dependant on the number of fragments chosen

Total particle numbers (sum of all particles, including minerals, proteins, organic particles, MPs, etc.) in 1 l of clean freshwater and drinking water are expected to be up to 10,000 particles, if a lower size limit of 5 μm is applied. If the lower size is set to 1 μm, more than one million particles can be detected (unpublished data of B. E. Oßmann). The sub-sample size has to be chosen with respect to the total particle number and the expected MP content to obtain representative results (see Fig. 2).

When analyzing particles on the filter, it is best to adhere to Gy’s theory of sampling, which deals with the errors that are introduced by incorrect sampling specifically for particles [91]. The total sampling error is dependent on two factors, the material heterogeneity and the sampling process [56]. In this context, Thaysen et al. show the spatial pattern of the particles distributed over the filter impacts the different window selection schemes [92].

At this stage, it is advisable to use one of four different models: (1) “total surface/all particles,” (2) random, (3) “cake,” or (4) “snail” models (see Fig. 2). All models rely on an automated particle localization routine (open-source or commercial software).

If it is feasible to measure every single fragment (particles and fibers) of the sample, this should be done, as any form of sub-sampling will introduce an error. This model is called “total surface/all particles.” For FPA-FTIR imaging, this may be possible for fragments down to 10–20 μm. For single particle measurement (e.g., for RM), this is appropriate, if the fragment count is below 1000 (or 500 for practical reasons, 7000 best practice). If this number of fragments is exceeded, sub-sampling is necessary to reduce measurement time. This is similar to a second sampling step, where all fragments on the filter represent the lot. The objective is to make a correct selection. According to the theory of sampling [91], all fragments of the lot must have the same probability of being selected as part of the sub-sample. Therefore, no bias is introduced into the process. The investigated fragments are in the size range of 10–500 μm (or 5–500 μm) and might agglomerate depending on their physical properties. Thus, they may not have an even, random distribution on the filter (Fig. 2: inhomogeneous particle distribution, i.e., grouping of particles according to material type is represented in blue, yellow, and green). This effect is expected to be negligible for fragments at the lower end of the size range, but will have a great effect on fragments at the upper end. A homogenization step for the fragments on the filter would be required. Usually, homogenization implies the reduction of grain size or thorough mixing, none of which is applicable to particles deposited on a filter. Therefore, a virtual mixing is required. The virtual mixing can be achieved by applying three types of sub-sampling. The most advisable sub-sampling strategy is the random model. This corresponds to a random selection of particles, without replacement, from the original lot (all particles on the filter) to form the sub-sample. However, random sampling is not applicable with every software. Another possibility is to take multiple “cake” slices (see Fig. 2). The “cake” model is suitable for taking into account an inhomogeneous distribution of particles between the center and the edge of the filter. In order to represent also inhomogeneous distribution of particles among different filter areas (e.g., filter halves), multiple “cake” slices must be analyzed. To avoid a grouping and segregation error, all large fragments (> 50 μm) should be measured (up to a number of 1000 or 500 for practical reasons). Particles down to a size of 5 μm should be analyzed on “cake” slices that, in sum, cover at least 20% of the total filter area. If even smaller particles (1– 5 μm) are targeted, particles in this size range should be analyzed separately, using an objective with higher magnification. In previous works, 4% of the filter area have been investigated for analyzing this size class [32]. As a suitable alternative, the “snail” model (also called “helix” model, see Fig. 2) can be applied. It uses boxes that are distributed along a spiral to represent all areas of the filter (center and edges). In contrast to the “cake” model, it applies many small boxes. Reducing the box size, while increasing the box number, contributes to a virtual mixing of the fragments and, therefore, reduces the grouping and segregation error [93]. For this model, the same rules for filter area and fragment number apply, as for the “cake” model. The ultimate reduction in box size and increase in box number will finally result in the most advisable model, the random model. The random model can be applied in two forms: a full random sampling (selection of particles regardless of their size) or a stratified random sampling in different size classes (e.g., 1000 particles ≥ 50 μm and 7000 particles < 50 μm). The random model ensures an unbiased selection, and the error can be estimated with Eq. 1 based on the final measured MP content [87].

1 $$ n\ge \frac{\left.P\Big(1-P\right)}{\frac{e^2}{\sigma^2}+\frac{\left.P\Big(1-P\right)}{N}} $$

**Table:** 

Variable	Symbol	Required information
Confidence interval	*σ* = 1.65	For 90%
Total number of particles	*N*	Particle count from detection
Estimated MP content	*P*	From prior experiments/literature
Margin of error	*E*	Inherent to research question
Sample size	*n*	Determined by image analysis

**Fig. 3:**
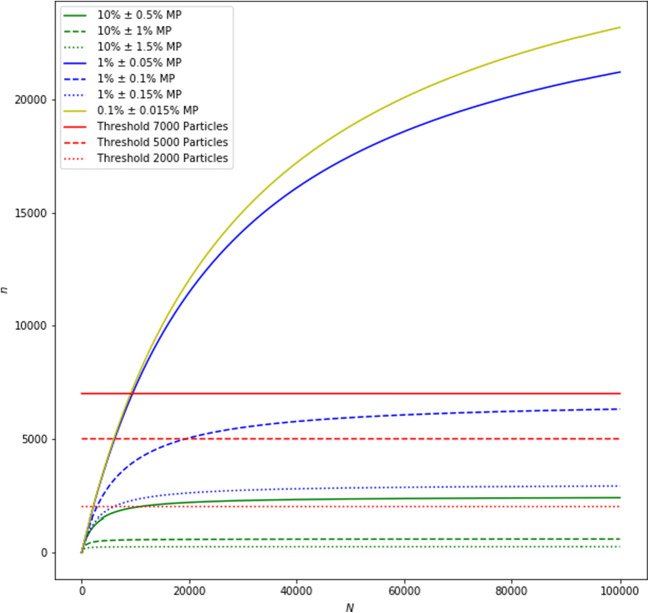
Dependence of sample size (*n*) on margin of error (*e* = 10%, 20% or 30%; e.g., 10% ± 0.5% MP, 10% ± 1% MP, or 10% ± 1.5% MP, resp.) and total number of fragments (*N*) for MP contents of 10% (green), 1% (blue), and 0.1% (yellow) for the random model, calculated based on Eq. 1 [87]. Sampling thresholds (*n* = 2000, 5000, and 7000) are marked in red

The error for different sample sizes and MP contents is displayed in Fig. 3 (the script and an executable file are added in ESM, section S2). The number of particles that need to be chemically identified for an effective quantification depends highly on the expected MP content and the desirable error margin. The goal is to minimize the number of randomly selected particles in the sub-sample to save valuable measurement time. As can be seen in Figure 3, 7000 particles, which correspond to approx. 48 h measurement at 20 s acquisition time per particle (e.g., over the weekend, or approx. 12 h for 5 s per acquisition time per particle) is suitable for most MP contents and error margins (all green and blue lines except solid blue). If the MP proportion should in fact be smaller than expected (yellow solid line), the measurement of further particles is necessary. This is easy to accomplish as additional particles, which were not measured previously, can be randomly selected from the sample and measured to augment the original sample size. Should the error margin be set too ambitiously (blue solid line) there are two options: (1) measure more particles to be within the error margin or (2) recalculate the error margin and accept a greater error margin than originally desired. In case a very high MP content, e.g., 10% is expected, a threshold close to 2000 particles may already suffice (all green lines). A threshold of 7000 particles is sufficient to ensure a representative sampling in most cases. However, if the MP content is very low (e.g., 0.1%, see yellow line in Fig. 3), only filters with small numbers of deposited particles can be measured quantitatively. This underlines the importance of sample preparation, i.e., removal of inorganic and organic matrices in order to increase the ratio of plastic to non-plastic fragments.

While Gy’s theory of sampling and the work of Anger and von der Esch et al. [87] provide means to estimate sub-sampling errors theoretically, Brandt et al. have investigated the errors introduced by different sub-sampling sizes and models based on actual samples [94]. For that purpose, they have chosen fully analyzed filters from 27 environmental samples (rain water, riverine surface water, riverine sediment or beach sand, wastewater sludge) and performed artificial sub-samplings according to the random model (stratified and full random) as introduced here, and different area box-placement strategies, among others, one that resembles the snail model. The environmental samples contained between 1500 and 33,000 particles, and had MP contents between 0.5 and 4.7%. Brandt et al. [94] found that none of the tested sub-sampling models clearly outperformed the others, apart from edge scenarios (i.e., area box-placement models perform worse for filters with < 2000 particles and inhomogeneous particle distribution). Furthermore, they recommended measuring at least 7000 particles or 50% of the particles using the random models, or 50% of the filter area in box-placement models, if the sub-sampling error is to be kept below ± 20% (which corresponds to e = 40% in the approach of Anger and von der Esch et al. [87]). Concerning the random models, the advice to measure 7000 particles lines out with the recommendations discussed before, but measuring 50% of the particles on a filter is, in part, stricter than Figure 2 would suggest. However, Brandt et al. [94] found that the standard deviation of the sub-sampling error increases considerably with decreasing measurement fraction, while the graphs in Figure 2 rely on the standard deviation of the normal distribution. Therefore, Brandt et al. tailored their recommendation to meet the < 20% sub-sampling error requirement with more security. Furthermore, it has to be noted that discussed random sub-sampling strategy is well applicable for particles larger than 5 – 10 μm, where all particles on the filter can be detected, the minimal sample size can be calculated, and errors can be quantified. In contrast, smaller particles might not all be detected on the entire filter, demanding a window-based analysis. For this purpose, a bootstrap method has been recently proposed by Schwaferts et al. [95] to provide an error quantification with confidence intervals from the available window data. In this context, different window selection schemes have been evaluated and there is a clear recommendation to employ random (rather than systematically placed) window locations with many small rather than few larger windows.

In summary, if applicable and technically possible, all particles on the filter should be measured (“total surface/all particles”). Otherwise, a sub-sampling strategy has to be applied. The most recommendable sub-sampling strategy is the random model, because it is not susceptible to inhomogeneous particle distributions on a filter. Only if a random model cannot be realized (e.g., due to technical reasons) the “cake” model or area box-placement models such as the “snail” model should be applied. In that case, sampling of small but numerous boxes or “cake slices” is always preferable.

While for the random model, theoretical error calculations have been proposed, they must still be developed for area box-placement models (e.g., “cake” and “snail”), which will be especially challenging for heterogeneous samples. Further research is also needed for validating any of these models using actual, fully measured samples and artificial sub-sampling.

### Analytical outlook

In addition to the commonly used IR and Raman approaches previously described, some more recently developed applications/techniques show promising potential for the analysis of micro- and nanoplastic pollution in the future. Theoretically, μ-(FT)IR and RM are limited to minimal particle sizes of about 10 μm resp. 300 nm [31, 64, 77, 86, 87]. Recently, the potential use of confocal RM for particle sizes even below this limit (down to 100 nm) was demonstrated [96, 97]. Small micro- and nanoplastic particles (down to a size of 50 nm) can be analyzed directly in liquids via a combination of RM and optical tweezers (OT) [98]. Moreover, separation, characterization, and chemical identification of these particles can be realized via online coupling of field-flow fractionation and RM enabled by OT [99]. Furthermore, IR spectroscopy can be combined with other techniques such as atomic force microscopy (AFM) to lower its spatial resolution to about 20–50 nm [100, 101], thus permitting the analysis of plastic particles at nanoscale. Surface-enhanced Raman spectroscopy (SERS) may play a role in future applications as recently shown by Zhou et al. for PS nanoplastics as small as 50 nm [102]. The spatial resolution could be improved in the future by the application of tip-enhanced Raman spectroscopy (TERS) [103]. However, the applicability of these methods for different polymer types and on real samples has still to be demonstrated. Especially organic residues, such as humic acids, which might not be entirely removable, might cause interferences [9].

## Data processing

Spectroscopic methods provide information not only on chemical identity but also on particle characteristics (e.g., size, shape, color). Thus, data processing has to evaluate large sets of spectral as well as particle-related data. Due to the large number of particles, we suggest using computational, highly automated solutions for time-efficient analysis. Instrument manufacturers are only beginning to offer particle analyzer programs that not only allow for the acquisition of large numbers of spectra automatically but also provide high-performance data-processing tools. Meanwhile, research groups have developed their own data-processing routines based on commercial software [104]. Furthermore, open-source solutions for both μ-FTIR imaging (e.g., siMPle [79]) and RM (*TUM-ParticleTyper* [62]; see “Raman microspectroscopy”) or for RM and μ-FTIR spectroscopy (GEPARD [49]; see “Raman microspectroscopy”) are used. In the following, general guidelines for spectra evaluation and reporting for any data-processing approach are described.

Regardless of the applied measurement method, large numbers of spectra are generated. They must be evaluated while assuring both correct identification and time-efficient processing. Ideally, the software tools do not only ensure equal treatment of all spectra of a dataset, but they also are fast and stable when processing large datasets and provide convenient formats of data output as well as (easy to use) tools for the operator to evaluate and double-check the identification of the automatically acquired spectra.

There are various approaches to the evaluation of spectra, such as correlation to spectral databases [29, 74, 105], model-based classification [104], or descriptive methods (e.g., multivariate curve resolution-alternating least squares (MCR-ALS) and independent component analysis (ICA)) (El Rakwe et al., in prep.). Database searches rely on similarity measures, such as the absolute distance, the Euclidean distance, least squares [106], or the Pearson correlation coefficient to find an unknown spectrum’s match in a database. For large datasets, the correlation of spectra to databases can become very slow or require high computational power. Reducing the number of spectra in the database can reduce the processing load. Nevertheless, database searches can reach method-intrinsic performance limits, especially for spectra from environmental samples with low signal-to-noise ratios or other deviations from the ideal polymer spectra. Alternatively, training a set of chemometric classifiers, i.e., of random decision forest (RDF) classifiers, in combination with applying spectral descriptors for data preprocessing was proposed [64, 104]. Classifiers are complex algorithms that sort data into categories of information (classes). They learn to predict the class affiliations of unknown data from labeled training data. For the identification of polymer spectra, a training dataset is used which is comprised of a sufficient number of representatives from all the polymer classes that shall be detected. For further details, reference may be made to Hufnagl et al. [104]. Spectral classifiers or descriptive methods can reduce the computational demands, but their implementation is not trivial and becomes more challenging as the number of substance classes increases.

To date, the most common approach is the correlation of spectra to databases. During this process, the search parameters (e.g., raw or derivative spectra, spectral range, model of comparison [106]), data treatment (e.g., background correction, spectral normalization), and spectral quality can significantly influence the results [107]. Likely, the best control of whether a polymer spectrum has been identified correctly is an experienced spectroscopist’s evaluation. Such a manual QC can, however, be time-consuming due to the large number of spectra. As an alternative, quality criteria for the automated spectral identification can be defined. Database software frequently provides a quantitative criterion for the similarity of a spectrum and a database spectrum, which is often referred to as the hit quality index (HQI). Using a HQI of 70% as a threshold for accepting a result as correct has been suggested [68, 108]. Beside this absolute value, the HQI difference (ΔHQI) of the first to the second-best hit can be consulted, reflecting especially the selectivity of the correlation as pointed out by Renner et al. [109]. For this purpose, two suitable approaches to define a threshold exist: (i) ΔHQI ≥ 10% or (ii) ΔHQI ≥ HQI × 0.1. However, both concepts will not allow distinguishing between similar polymers or subclasses of polymers like low and high density polyethylene (LDPE and HDPE) or polyamide 6 and polyamide 6.6 (PA 6 and PA 6.6) making re-evaluation by manual inspection necessary [109–111].

Furthermore, it has to be underlined that HQI values generated with different software might not be comparable, as the suppliers have implemented varying algorithms. Furthermore, the absolute HQI value depends on the quality of spectra and the type of sample (reference material or environmental sample). Consequently, the data pretreatment, the applied correlation algorithm, as well as the database to which the spectra were correlated needs to be reported in studies [109]. As the HQI depends on many different factors, a general numerical value cannot be defined as a quality criterion. If a laboratory chooses to use only the HQI as quality criterion, determination and validation of the HQI thresholds with respect to the instrumental capabilities and the analyzed sample types is obligatory.

To the best of our knowledge, there are no standard operation protocols on how to accomplish validation of HQI thresholds, but a thought experiment based on statistical hypothesis tests could be considered and is described in the ESM, section S3. It should be noted that it has not been tested in routine analysis, so its purpose is to provide the reader with a suggestion on how to approach the task. We strongly encourage the laboratories to thoroughly test and modify the approach and to share their findings.

Current experience is not yet sufficient to provide an assurance to readers that statistical hypothesis tests allow a failure-safe determination of a correct HQI but we encourage verifying one’s HQI thresholds with a suitable approach instead of picking an unsubstantiated number.

If approaches other than correlating spectra to databases are used, these recognition models have to be validated and reported just as the HQI values described above.

As a standard set for spectral identification, we suggest to include the following main polymers: polyethylene (PE), poly (ethylene terephthalate) (PET), PP, PS, PC, poly (vinyl chloride) (PVC), poly (methyl methacrylate) (PMMA), PTFE, polyamide (PA, nylon), and polyurethanes (PU). The latter can possess a variety of side groups depending on the monomers they are made from, resulting in differing IR and Raman spectra. It is thus recommended that different kinds of PU are included into the set of standard polymers. Since spectra of polymers can differ significantly due to weathering of MPs, it is also advisable to include spectra of aged polymers in the database [112]. Each laboratory is furthermore recommended to augment this set with further polymers (e.g., acrylonitrile butadiene styrene (ABS), natural rubber, copolymers) as well as non-synthetic and/or non-polymeric materials that can commonly occur in samples (e.g., amorphous carbon, cellulose, proteins, inorganic materials such as quartz, and other minerals). As stated in “Raman microspectroscopy,” Raman spectroscopy can identify additives such as pigments and dyes. In the case of these colorants, their typically sharp peaks can be of higher intensity than polymer peaks. Therefore, optimized measurement parameters have to be applied for identification of colorants/pigments and polymers. In order to recognize these potentially polymeric particles, the inclusion of a library of common dyes for polymers can be useful. Spectral libraries are commercially available, but many software tools for database search allow the creation of personalized libraries. This can be advantageous as database and sample spectra are obtained with the same instrument, and spectra of potential contaminants occurring just in a certain lab can be included [80]. Very recently, Cowger et al. developed a new open-source library (Open Specy) for MP identification for both measurement techniques, RM and IR. Regarding particle identification, it is fully comparable to commercial software. Users can upload and share their own spectra with the community, so that the library is continuously growing and improving [113].

The operator must pay attention to distinguish the spectra of PA and proteins. For both techniques, they can be distinguished well when comparing high-quality spectra. However, as spectra from environmental samples are often distorted, identification has to rely on the most prominent peaks, which are similar both in Raman and in (FT)IR spectroscopy. Detailed information on this difficulty can be found in the ESM, section S1. Molecules containing long carbon chains can have very similar IR and/or Raman spectra. This is the case, for example, between PE and stearates (e.g., found in latex gloves and used as food additives), stearamides (e.g., used as polymer additives and slipping agents) or sodium dodecyl sulfate (e.g., used as detergent) ( [45] and unpublished data, N. Benismail).

Overall, each laboratory is encouraged to augment these data-processing recommendations according to their needs. Details of the determination of the particle properties and the spectral identification must be reported as stated in “Ways of reporting results and valuable information.”

## Method validation and quality controls

In analytical chemistry, method performance is assured by the determination of QA and QC parameters. Both together prove that a given method is effective and reliable. These include the calculation of recovery rates, repeatability, reproducibility, limits of detection and quantification (LOD and LOQ), and blank contribution as well as overall robustness. In addition, ILC studies are necessary to check accuracy and precision of the methods used by different laboratories (see also Figure 1).

Method validation is an analytical challenge, especially in the case of MP analysis, and the following difficulties must be taken into account: Unlike other conventional contaminants, MPs are not dissolved in aqueous samples, but are present in particulate form. Therefore, they are not homogenously distributed and up to now, suitable certified reference materials are not available. As the expected concentration and size of MPs in clean water are very low, a mass-based approach cannot be regarded as promising (see “Introduction”). A first international ILC study on MPs in water has been initiated by the Joint Research Centre (JRC) of the European Union [21], but at the time of writing, the results and conclusions are still pending. They can be a basis for further international proficiency tests (PT).

In most studies on MPs, QA/QC data include solely the analysis of blanks for control of sample contamination. Wang et al. used pre-filtered drinking water as blank sample. After filtration through aluminum oxide filters (pore size 0.22 μm) and analysis with SEM and RM, the blank contribution was < 5% of MP abundance [114]. Pivokonsky et al. used water of HPLC-grade and analyzed particles > 10 μm with FTIR spectroscopy and particles < 10 μm by RM. They also report a blank contribution < 5% [34]. On the other hand, Zhang et al. indicated that there was no blank contribution when filtering 1 l ultrapure water through nitrocellulose filters (pore size 0.45 μm) with subsequent ATR-μ-FTIR analysis [115]. Contrarily, filtration of 150 l of pre-filtered (with 3-μm stainless steel cartridge filters) drinking water using aluminum oxide filters (pore size 0.2 μm) resulted in a blank contribution of 45 ± 22 fibers of different colors [59]. Schymanski et al. reported a blank contribution of 14 ± 13 MPs (size down to 5 μm) in 1 l ultrapure water (system fed with deionized water, end filter with 0.22 μm pore size) [33]. Predominant polymers detected were PET, PE, PS, and PP. When analyzing particles down to a size of 1 μm, even blank values of 384 ± 468 MPs/l are common [32]. Furthermore, several authors report the procedures to prevent sample contamination during MP analysis [34, 52, 116, 117].

However, published papers seldom provide QA/QC data regarding efficiency, repeatability, and reproducibility or on LOD/LOQ. As methods are not sufficiently validated, quantitative analysis is affected by the analytical technique used and its capacity to detect and identify MPs with confidence. So far, there are still important analytical limitations regarding the filtration step (choice of filters and sample volume) and the detection and the identification potential of analytical methods (IR spectroscopy/RM). In addition, there is still a certain subjectivity for data evaluation.

Overall, there is an urgent need to perform analysis under QA/QC management. This will lead to more uniform and comparable data. QA/QC measures of sampling, sample preparation, and detection will permit the development of a more realistic picture of MP occurrence, especially for small-sized MPs [20, 118].

One of the main steps of the QA/QC system is to characterize the methodology and to perform the initial validation of the method. For this specific methodology, we propose to assess the following important steps:

I. Verification of the size measurement capability of the equipment (minimum requirement)
II. Verification of qualitative polymer identification at the claimed minimal size (minimum requirement)
III. Determination of the LOD (minimum requirement)
IV. Verification of the recovery rates of the entire method (best practice)
V. Participation at interlaboratory comparisons and proficiency tests
I. Verification of size measurement

To prove that particles are being assigned correct size values, the size calibration of the particle detection should be performed with, for example, certified size standards or engraved plates/filters. Uncertified material can also be suitable, if the size is checked by optical microscopy. The particles with a certain size should be determined correctly within a tolerance of ± 10% [119], or, in case of imaging techniques which are tied to a certain pixel resolution, within a tolerance of ± 1 pixel [64].

II. Verification of qualitative polymer identification at minimal size

Verification of qualitative polymer identification should be performed using the most commonly applied polymers (PE, PET, PC, PP, PVC, PS, etc.) and analyzing at the claimed minimal detectable size. For validation, MPs from real samples (with previously identified polymer type), commercially available polymer standards [61], or in-house-produced reference polymer particles [64, 69] may be used. Very recently, the JRC published their method on how they produced reference material for the above-mentioned ILC [120]. The lab is required to achieve good-quality spectra. If an automated database algorithm for polymer identification is used, acceptable quality indices for the library match should be documented (see HQI, “Data processing”). It is highly recommended to check whether the expected polymers, e.g., the plastic container/cap of bottled water can be recognized by the analysis. Otherwise, these spectra have to be added to the database or the analysis parameters must be adapted.

III. Determination of the limit of detection

The LOD is a global value demonstrating the capability of the laboratory to master its scientific equipment, filtration systems, but also the potential contamination of samples with MPs coming from the lab environment, air, operator, or lab materials [20].

The most challenging QA/QC step is the verification of the quantitative MP count. Usually, the LOD and LOQ are common parameters in analytical chemistry to describe the sensitivity of a method. The LOD describes the minimum amount of an analyte that can be detected but not necessarily quantified [121]. The smallest accurately quantifiable amount of an analyte is described by the LOQ. There are different methods to determine or calculate the LOD and LOQ for calibratable techniques of dissolved substances, but these are not applicable to solid particles like MPs, which cannot be diluted. In this case, the LOD of the method, which describes the smallest amount of detectable MPs in the sample, can be determined as the mean of blank samples plus the threefold standard deviation (minimum requirement; see Eq. 4). The determination of LOD based on blank values is in agreement with several recently published papers on microplastic analysis by μ-FTIR [53, 64, 122, 123]. We suggest using 10 blank samples to initially calculate the LOD. Ideally, this should be done for every particle size category/range that is given in the report with each LOD being expressed in relation to the size range values (e.g., LOD [5 μm] = 8 MPs). In practice, quoting the LOD for the smallest size class is sufficient.

4 $$ LOD={mean}_{10\  blanks}+3\ast {s}_{10\  blanks} $$

Verification has to include the entire analytical process and has to take into account different polymers present in (blank) samples. Thus, LOD may be expressed as the undifferentiated sum of all polymers. For detailed scientific studies, polymer-specific LODs can be more advisable.

Additionally, when the experience in MP analytics has grown, the LOQ should be calculated and reported as the mean of 10 blanks adding the tenfold standard deviation (best practice). This will provide accurate qualitative data for the contamination of water samples.

IV. Verification of the recovery rate of the entire method

As long as there is no certified reference material and the possibility to prove the quality of the method with an ILC, the accuracy of a method is not fully provable [124]. Therefore, the recovery rates should be calculated based on the analysis of spiked samples (even with different levels of MPs) in the best practice approach [20]. Enders et al. determined the recovery rate of their entire extraction procedure with a new modified separation funnel for particle-rich samples with 60–80 fluorescent PE particles in the size range between 125 and 150 μm [28]. They reported a recovery rate of 78 ± 6%. Although they published a protocol to extract MPs between 10 μm and 5 mm from environmental samples, they did not determine the recovery rate for particles between 10–125 μm and 150 μm–5 mm. For a best practice routine, recovery rates should be determined for the whole particle size range targeted in the analysis. However, exact spiking of particle numbers with sizes < 100 μm is still a challenge.

Analysis of standards for cytometers can be an alternative, if it is proven, that the system recognizes the spherical shapes correctly [119]. Another possibility is to use commercially available MPs of different sizes [61] or to produce own reference materials [69]. Applying the latter method, dispersed secondary MP reference particles can be generated via ultrasonic treatment. Nevertheless, there is still a high need for reference materials of different polymer types with a defined number, size or size range, and shape (incl. spheres, irregular fragments, films, and fibers). These would enable comprehensive validation and ILC studies for different methods.

To simplify, one can determine the recovery rate of the whole method with just one kind of polymer, assuming a similar behavior of main polymers during preparation and filtration steps of the method.

Once the important characteristics of the method are verified, the lab should complete the quality management of the method by systematically preparing blank (process) controls in each series of samples. These blank samples are necessary to demonstrate the correct sample handling and to ensure the validity of the results (see “Laboratory blanks”).

V. Participation at interlaboratory comparisons and proficiency tests

An important step in the generalized acceptance of analytical methods is their testing through ILC. Currently, there are no widely accepted standardized methods and very few reports of ILC or PT undertaken to evaluate potential analytical MP procedures. In literature, a number of projects or consortia have reported the organization of restricted, internal ILC [23, 27, 125]. However, with the exception of the work of Isobe [126], where MP particles ranging from 0.4 to 5.7 mm were analyzed, the detailed outcome of the results is not openly available. With regard to ILC or PT studies, where participation is freely accessible to a wide range of laboratories, there are no fully documented studies at this time. But, recent initiatives by QUASIMEME [22] and subsequently JRC [21] provide examples of ongoing trials. These two examples neither propose predefined methods or protocols, but rather supply participating laboratories with one or more types of (as far as possible homogeneous and stable) MP-containing samples. These samples are analyzed by methods and procedures, which the participants judge appropriate to provide the requested data, e.g., plastic identity, particle number, and particle size. In both cases, the results and experience gained during the trials are intended to aid harmonization of methodologies while, in the latter case, an additional aim is to undertake the first steps toward the development and evaluation of potential MP reference standards.

While the lack of information from ILC studies is limiting method development and harmonization, there is a growing public and political awareness of MPs, which is driving legislation. It will require standardized methods, e.g., the recast of the EU Drinking Water Directive [127]. These legal drivers will increase the need for methods to be subjected to more rigorous QC tools, such as ILC and PT exercises. Quality control will in turn require general access to fit-for-purpose reference materials. The development of such materials will be a technically complex and time-consuming process, which presents many challenging issues. Firstly, defining how a reference material should be designed is a key issue given the huge range of polymer types, additives, shapes, sizes, and concentrations, which can be present in the many types of background matrices. For some applications, such as MPs in drinking water, the composition (regarding the variety of polymer types and size range and shape of MPs) may be simpler. For other sample types, such as sediments or municipal wastewater, test samples will be much more complex to define representatively. Secondly, it is likely that widely available standards would incorporate artificially manufactured MPs (e.g., mechanically milled or custom synthesized). These may present behavioral variations (agglomeration state, adhesion to filters and laboratory equipment) resulting from their surface properties (hydrophobicity, charge), which are not typical for MPs from real (environmental) samples. These differences in the behavior of MP fragments may influence overall sample recovery and, in the case of agglomeration, compromise particle counting results. One possibility to overcome these limitations is to generate dispersed secondary MP reference particles via ultrasound treatment [69]. Finally, verifying adequate homogeneity across sample batches and testing temporal stability will need reliable and reproducible procedures prior to performing more extended ILC trials. These will be necessary to assign consensus values (particle number, mass, size distribution, etc.) to the standards.

## Ways of reporting results and valuable information

A report of MP analysis must contain all parameters of the sample, sample treatment (description of filtration, filter type, and pore sizes), and the analytical methods [17]. For the calculation of the total number of microplastics in the sample, subtracting the laboratory blank value of the sample series or the limit of reporting from the sample results is not recommended. The laboratory should indicate the value of LOD with the results. For (FT)IR spectroscopy, these are measurement mode, use of FPA detectors, spectral range, spectral resolution, and number of co-added spectra. Parameters for RM that need to be reported are as follows: optical imaging mode (bright or dark field), spatial resolution of the image (pixel size), microscopic objective (magnification and numerical aperture) for Raman measurement, confocality, wavelength of excitation laser and laser power at the sample, grating, focal length of the spectrometer, exposure time, and the number of co-added spectra. For both spectroscopic methods, the description and validation of the spectral identification routine have to be included. For communication of the analytical results, a report on the MP content of a sample obtained with spectroscopic methods must contain the following results:

- Total number of particles in the sample or sub-sample (see “Models for sub-sampling of particles deposited on a filter,” if possible to obtain this value with the instrument used)
  - In case of sub-sampling additionally: analyzed area of the filter (%) or statistical percentage of analyzed particles on the total number of particles (%)
- Number of particles analyzed (obtained by measurement)
- LOD: preferably size-dependent (see “Method validation and quality controls” for more details)
- Number of total MPs identified (obtained by extrapolation or measurement)
  - By type of polymer
  - By size ranges

At present, it is common practice to report MP numbers in predefined size classes, i.e., in a binned form [10, 54, 81, 128, 129]. By this, the details on the size of each MP detected in samples get lost. However, these details are relevant for risk assessment. As the non-alignment of methods and reporting hinders risk evaluation [130], future studies should at least use the same size classes for binning, as proposed in Table 1 in the section on analysis: 1–5 μm, 5–10 μm, 10–20 μm, 20–50 μm, 50–100 μm, 100–500 μm, and > 500 μm. Nevertheless, the future best practice should include the reporting of details on the particle size of all detected MPs, e.g., in the form of supplementary data or at least on demand.

As additional information or upon request, the report might contain:

- Details about blank samples (number, type of polymer, size range)
- Shape (e.g., fragment, fiber, sphere) and color of particles
- (Microscopic) images of measured filters
- Number of (potential) MPs not included in the minimum set of polymer classes proposed in “Data processing” (e.g., colored polymers, pigment or dye particles) sorted by type or by size range, shape, color, etc.
- Total number of non-plastic particles identified (e.g., minerals, proteins, cellulose) sorted by type or size range, shape, color, etc.
- Total number of non-identified particles
- Software and routines used for
  - Determining particle properties
  - Spectra processing
  - Spectral identification; in the case of database search information about the database (commercially, homemade); if a HQI was used, the algorithm used and how the threshold was determined

Laboratories must only express quantitative results, if they exceed the LOD at the size limit of the method. Particles that are, e.g., smaller than the filter pores, can be found on a filter nevertheless, if deposited between the pores. The proportion of such particles retained is unknown, and thus, their number is not representative for the sample. Furthermore, blanks and possible cross-contaminations have not been evaluated for particles smaller than the size limit. However, these numbers can be of interest as an indicator of the amount of smaller particles in the sample. Thus, if a laboratory provides these quantitative values, it must demonstrate unambiguously that any of the particles counted are intrinsic to the sample in order to eliminate any risk of false-positive results, and state that this number is not representative for the sample.

## Discussion and conclusions

The lack of harmonized methods and analytical standard substances and the difficulty to validate methods for the determination of MPs in clean water have led to highly variable and even sometimes contradictory data [15]. The proposed quality criteria by Koelmans and colleagues include the sampling method, sample size, sample processing and storage, laboratory preparation, clean air conditions, positive and negative controls, sample treatment, and polymer identification. The present consensus paper discusses and sums up details regarding the most important spectroscopic methods that can be used for MP analysis in clean water. All of the above-mentioned quality criteria were integrated in this guideline (see Table 1). It allows the reader to compare and evaluate existing studies. Furthermore, the guidelines can be used to better understand and thus make a more advantageous choice when setting up MP research studies. Given best practice approaches will contribute to a better harmonization of analytical methods for MP analysis in clean water samples down to 1 μm. A schematic overview of important precautions for MP analysis and sampling advices are given in Figure 1.

All these elements are intended to support the standardization processes throughout the different normalization committees. While this consensus paper from twelve European analytical laboratories and institutions has concentrated on (FT)IR/RM methods, for the purpose of monitoring as well as gaining a more comprehensive knowledge on MP contamination in food, water, air, and environmental samples, both spectroscopic and thermo-analytical methods are required. Therefore, it will also be important that a similar consideration be given to harmonizing thermo-analytical methods for MP detection. Above all, an ongoing exchange of scientists and laboratories, ILC studies with certified polymer standards and coordinating structures are required. These will pave the way to enable progress in the harmonization and standardization of MP detection and to allow for representative and reliable MP analysis in different environmental and food samples.

## Supplementary Information

ESM 1 (PDF 419 kb)

ESM 2 (PDF 379 kb)

## Abbreviations

ABS: Acrylonitrile butadiene styrene
AFM: Atomic force microscopy
ATR: Attenuated total reflection
FPA: Focal plane array
FTIR: Fourier transform infrared
HQI: Hit quality index
ICA: Independent component analysis
ILC: Interlaboratory comparison
IR: Infrared
JRC: Joint Research Centre
LDIR: Laser direct infrared imaging
LOD: Limit of detection
LOQ: Limit of quantification
MCR-ALS: Multivariate curve resolution-alternating least squares
μ-FTIR: Micro-FTIR
MP(s): Microplastic(s)
OT: Optical tweezers
PA: Polyamide
PC: Polycarbonate
PE: Polyethylene
PET: Poly(ethylene terephthalate)
PMMA: Poly(methyl methacrylate)
PP: Polypropylene
PU: Polyurethane
PS: Polystyrene
PT: Proficiency tests
PTFE: Polytetrafluoroethylene
PVC: Poly(vinyl chloride)
QA: Quality assurance
QC: Quality control
QCL-IR: Quantum cascade laser-based infrared
RDF: Random decision forest
RM: Micro-Raman spectroscopy
SEM: Scanning electron microscopy
SERS: Surface-enhanced Raman spectroscopy
TERS: Tip-enhanced Raman spectroscopy
WHO: World Health Organization
