# Supplementary material for: Analysis of microplastics in drinking water and other clean water samples with micro-Raman and micro-infrared spectroscopy: minimum requirements and best practice guidelines
Source: Anal Bioanal Chem. 2021 Jul 20;413(24):5969–94. doi: 10.1007/s00216-021-03498-y (PMC8440246; doi:10.1007/s00216-021-03498-y)
Supplement: Supplementary file 1 — (PDF 419 kb) [file 216_2021_3498_MOESM1_ESM.pdf]

Supplementary Information

Analysis of microplastics in drinking water and other clean water samples with micro-Raman and micro-infrared spectroscopy: Minimum requirements and best practice guidelines

Darena Schymanski#, Barbara E. Oßmann#, Nizar Benismail, Kada Boukerma, Gerald Dallmann, Elisabeth von der Esch, Dieter Fischer, Franziska Fischer, Douglas Gilliland, Karl Glas, Thomas Hofmann, Andrea Käppler, Sílvia Lacorte, Julie Marco, Maria EL Rakwe, Jana Weisser, Cordula Witzig, Nicole Zumbülte, Natalia P. Ivleva*

# Shared first authorship

* Corresponding author: Email: natalia.ivleva@tum.de; Tel: +49 89 2180 78 119

S1

For IR, we propose to control visually the peak at 3300 cm^{-1} (amide A, ν(N-H)) that is broad for proteins and sharp for PA (Fig. S1a). Furthermore, in the PA spectrum the peaks around 1550 cm^{-1} (amide II, ν(C-N)+ δ(C-N)) and 1640 cm^{-1} (amide I, ν(C=O)) are both of strong intensity, which differs for proteins. In addition, a subsequent Raman measurement could verify the result. As in FTIR spectra, the peaks in the Raman spectra are sharper for polyamides than the modes of the same vibration in proteins (Fig. S1b). The most prominent case is again the peak of the N-H-stretching vibrations around 3300 cm^{-1}, but it is also visible for the amide I peak around 1640 cm^{-1} and the CH_2-bending peak around 1445 cm^{-1}. In contrast to IR, the amide II peak is very weak or invisible in Raman spectra. The position of the amide I band can also help differentiating. While it tends to be in the range of 1640 cm^{-1} and below for common polyamides [1], it tends to occur at higher wavenumbers above 1650 cm^{-1} for many proteins [2]. Especially in environmental samples, a poorer spectrum quality can render the peak sharpness an unreliable criterion. In that case, one can look for peaks that cannot stem from a polyamide but only from the amino acid residues of the proteins. Distinguishable peaks from the amino acids are not always present, but if they are, they provide a useful aid. A sharp, intense peak around 1000 cm^{-1}, for example, stems from the breathing vibration of the aromatic C6-ring of the amino acid phenylalanine. This peak occurs in all proteins in Fig. S1b. In addition, these spectra include a peak around 3060 cm^{-1} of the aromatic C-H stretching vibration, which does also not occur in PA6 (Nylon 6). A peak at 500 – 550 cm^{-1}, as occurring in the bovine serum albumin spectrum in Fig. S1b, is caused by the disulfide stretching vibration in cysteine-containing proteins [3]. For more suggestions of distinguishable Raman peaks of proteins, we refer to Rygula et al. 2013 [2].

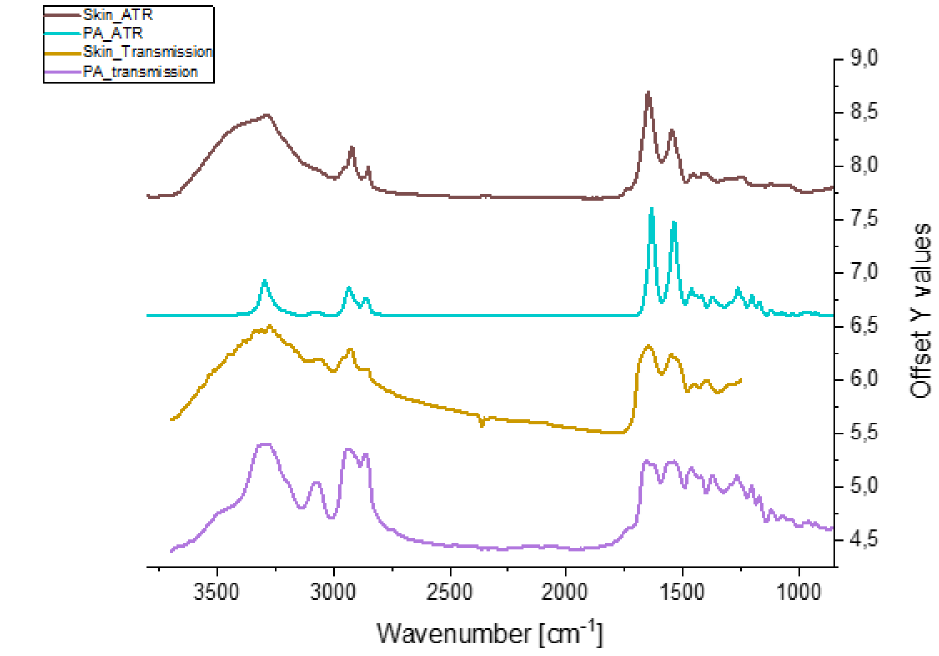

Fig. S1a. ATR- and FTIR spectra of polyamide and skin

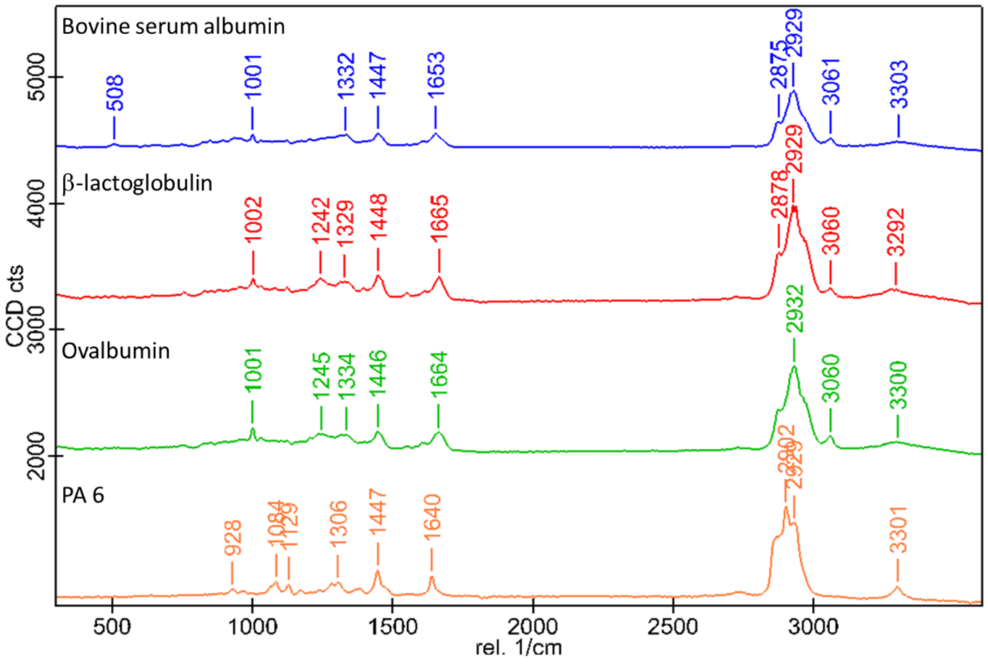

Fig. S1b. Raman spectra of the proteins bovine serum albumin, ß-lactoglobulin and ovalbumin and nylon 6 (PA 6) obtained with a WITec alpha 300R (532 nm, 10 mW, 1 s, 200 accumulations). The spectra are displayed with a y-offset for better visibility.

S2 see separate files

S3

To determine an approximate HQI threshold for a sample type, a fully automated analysis and a manual analysis of a whole dataset should be performed. Next, the number of particles per polymer type above a HQI of choice must be determined for both analysis results. Then, a statistical hypothesis test such as Pearson's chi-square test [4] must be performed to evaluate whether there is a statistically significant difference between the two results. Thereby, an

adequate level of confidence, e.g. a p-value of 0.05 must be chosen. If there is a statistically significant difference, the test should be repeated with an adjusted HQI, until the analysis results can be statistically assumed as identical. If there are too many false negatives (i.e., actual polymer spectra that are excluded because their HQI was below the HQI threshold), the HQI must be lowered. If the automatic results contain too many false positives (i.e., spectra falsely identified as polymers because the HQI threshold is too low), the HQI must be increased. The manual analysis for testing can be performed on representative subsamples instead of the whole samples for time efficiency. The determined HQI value should be validated by testing at least two more samples as described above. In addition, calculating an effect size (e.g., Phi, Cramer's V; [5]) can be helpful in assessing the consistency of the automatic and the manual analyses.

If the chi-square test is used, the number of polymer particles for each particle type in both, the automatic and the manual analysis results, must ideally be equal to or exceed n=5. As this is in accordance with the LOD of the analysis in many cases, the chi-square test is a suitable and easy to perform test. If the analyses cannot meet this or other boundary conditions of the chi-square test, a more suitable hypothesis test (e.g., Fisher's exact test) must be chosen.

The approach can be refined as fits the needs. That can involve an additional sorting of the particles into size categories. Another finding can be that a global HQI threshold for all polymer types cannot be defined. Then, HQI thresholds must be determined for subgroups of polymer types using the chi-square test, or even for single types by comparing the number of particles found in the manual and the automated analyses. Furthermore, the automated identification of a polymer type might fail completely. In that case, the test allows a reduction in the amount of data that has to be validated manually and thereby to saves time without giving up quality.

Current experience is not yet sufficient to provide an assurance to readers that statistical hypothesis tests allow a failure-safe determination of a correct HQI but we encourage verifying one's HQI thresholds with a suitable approach instead of picking an unsubstantiated number.

References

1. Hendra PJ, Maddams WF, Royaud I, Willis HA, Zichy V. The application of Fourier transform Raman spectroscopy to the identification and characterization of polyamides—I. Single number nylons. Spectrochimica Acta Part A: Molecular Spectroscopy. 1990; https://doi.org/10.1016/0584-8539(90)80031-S
2. Rygula A, Majzner K, Marzec KM, Kaczor A, Pilarczyk M, Baranska M. Raman spectroscopy of proteins: a review. J. Raman Spectrosc. 2013; https://doi.org/10.1002/jrs.4335
3. David C, Foley S, Enescu M. Protein S-S bridge reduction: a Raman and computational study of lysozyme interaction with TCEP. Phys Chem Chem Phys. 2009; https://doi.org/10.1039/b815492a
4. Pearson K. On the criterion that a given system of deviations from the probable in the case of a correlated system of variables is such that it can be reasonably supposed to have arisen from random sampling. The London, Edinburgh, and Dublin Philosophical Magazine and Journal of Science. 1900; https://doi.org/10.1080/14786440009463897
5. Cohen J. Statistical power analysis for the behavioral sciences: L. Erlbaum Associates; 1988.
