# Supplementary material for: Analysis of microplastics in drinking water and other clean water samples with micro-Raman and micro-infrared spectroscopy: minimum requirements and best practice guidelines
Source: Anal Bioanal Chem. 2021 Jul 20;413(24):5969–94. doi: 10.1007/s00216-021-03498-y (PMC8440246; doi:10.1007/s00216-021-03498-y)

S2

Theory_of_sampling_for microplastic_on_a_filter

March 22, 2021

1 Theory of sampling for Microplastic on a filter

Applying RM for the analysis of MP usually means analyzing single particles on a filter sequentially. The obvious question is: How many particles need to be analyzed to get a statistically meaningful result? The answer to this question strongly depends on sample matrix and success of sample preparation. Only for special cases if the total number of particles in the sample is low, as in, e.g., bottled water, it seems feasible to analyze all particles.

One solution to this problem is random sampling on a filter as proposed by Anger and von der Esch et al. 2018 https://doi.org/10.1016/j.trac.2018.10.010

The following code can be used to determine the minimum size of the subsample required for the analysis. Common subsample sizes have been included into the visualization as a benchmark. As will be shown by the simulation, it is very important to measure as many particles as possible. But there comes a point where the measurement effort for additional particles exceeds the improvement of the measurement error. This would be the optimal sample size if we had an ideal measurement setup. Since the measurement is not ideal this is the recommended minimal sample size.

How to reproduce the calculations:

To test the code run all cells. If modifications in the estimated microplastic content or error interval are desired, these can be changed in the cell below "Define the inputs". The cell below "Calculate the sample size and visualize the results:" then needs to be rerun to show the caluclation according to the new user input.

Test code by:

Elisabeth von der Esch elisabeth.esch@tum.de

[10]:
```
###Import nessecary modules
import pandas as pd
import numpy as np
import random
import matplotlib.pyplot as plt
import math
```

1.0.1 Define the inputs:

Inputs set by system:

Sigma value for prediction interval $\sigma = 1.65$ for \$90% \$ prediction interval

Total number of particles found on the filter through image processing N

Variable Inputs set by user:

Estimate of the MP fraction P

Margin of error e

Output:

Sample size/number of particles required n

[8]:
```
#THIS CELL CAN BE MODIFIED

# Define the estimated microplastic proportions P
P = 0.1 # 10% is the default this can be modified
e = P*0.1 # 10% ist the default error. E.g. a 20 % error would correspond to␣
 ↪P0*0.2
```

1.0.2 Calculate the sample size and visualize the results:

$$n \geq \frac{P \cdot (1-P)}{\frac{e^2}{\sigma^2} + \frac{P \cdot (1-P)}{N}}$$

The generated plot will show the minimum sample size for a total number of fragments between 1-100 000 according to the user specified estimated proportion of microplastic and the desirable margin of error defined in the cell above.

[9]:
```
#DO NOT MODIFY THIS CELL!!!!! But RUN THIS CELL TO SEE YOUR OUTPUT

# Calculate the sample size
s=1.65
n_0 = []
N_array = []

for N in range(1,100000):
    n = (P*(1-P))/((e**2/s**2)+(P*(1-P)/N))
    n_0.append(n)
    N_array.append(N)

# -----------------------------------------------------------
# Visualize your results

fig = plt.figure(figsize = (10,10))
ax = plt.axes()
plt.plot(N_array, n_0,'g-',  label=(''+ str(P*100)+'% \u00B1'+ str(round(e*100/
 ↪2 ,3)) +'% MP'))

ax.set(xlabel='N', ylabel='n',
```

```
      title='Dependence of samplesize (n) on microplastic content (P),␣
↪errormargin (e), and total number of fragments (N)');
plt.legend(bbox_to_anchor=(0., -0.2, 1., -.102), loc='lower left',ncol=2,␣
↪mode="expand", borderaxespad=0.)
plt.legend();
plt.show()
#save your plot
#plt.savefig('Sampling_error_random_model.png')
# End of TODO
# -----------------------------------------------------------
```

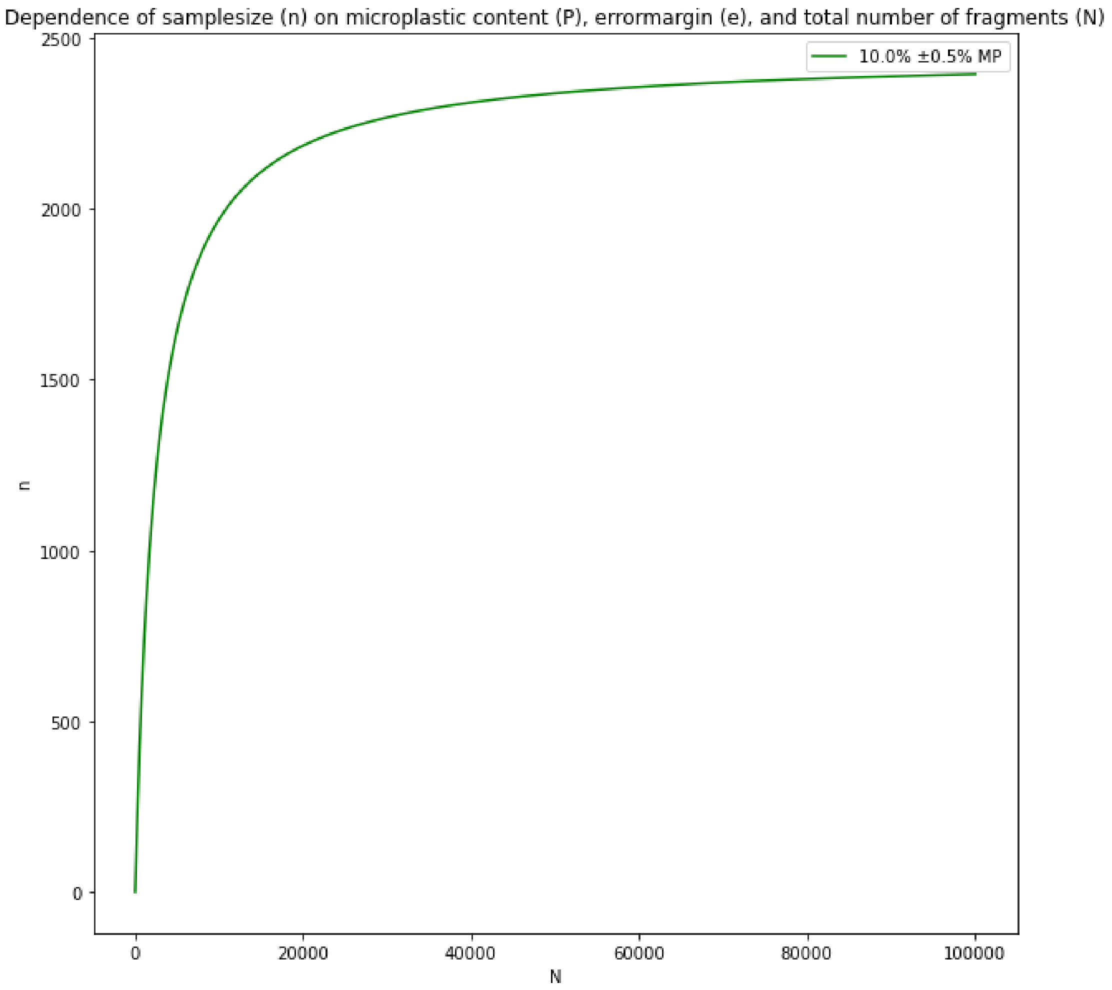

How to create the graph form the paper:

[11]:
```
# initialize variables
P1 = [0.1,0.01,0.001]
e1= [0.1,0.2,0.3]
```

```
s=1.65
N_array = []
n_1 = []

#caluclate sample size
for i in range (len(P1)):
    for j in range (len(e1)):
        for N in range(1,100000):
            n = (P1[i]*(1-P1[i]))/(((P1[i]*e1[j])**2/s**2)+(P1[i]*(1-P1[i])/N))
            n_1.append(n)
            N_array.append(N)

samplesize =[]
for i in range (9):
    n = n_1[i*99999:99999+i*99999]
    samplesize.append(n)

#calculate arrays for the thresholds
threshold1_array = []
threshold2_array = []
threshold3_array = []

for N in range(1,100000):
    threshold1 = 2500
    threshold1_array.append(threshold1)
    threshold2 = 5000
    threshold2_array.append(threshold2)
    threshold3 = 7000
    threshold3_array.append(threshold3)
```

[5]:
```
# -----------------------------------------------------------
# Visualize the results

fig = plt.figure(figsize = (10,10))
ax = plt.axes()
plt.plot(N_array[0:99999], samplesize[0],'g-',  label=' 10% \u00B1 0.5% MP')
plt.plot(N_array[0:99999], samplesize[1],'g--',  label=' 10% \u00B1 1% MP')
plt.plot(N_array[0:99999], samplesize[2],'g:',  label=' 10% \u00B1 1.5% MP')

plt.plot(N_array[0:99999], samplesize[3],'b-',  label=' 1% \u00B1 0.05%% MP')
plt.plot(N_array[0:99999], samplesize[4],'b--',  label=' 1% \u00B1 0.1% MP')
plt.plot(N_array[0:99999], samplesize[5],'b:',  label=' 1% \u00B1 0.15% MP')

plt.plot(N_array[0:99999], samplesize[8],'y-',  label=' 0.1% \u00B1 0.015% MP')

plt.plot(N_array[0:99999], threshold3_array ,'r-',  label=' Threshold 7000␣
 ↪Particles')
```

```
plt.plot(N_array[0:99999], threshold2_array ,'r--',  label=' Threshold 5000␣
↪Particles')
plt.plot(N_array[0:99999], threshold1_array ,'r:',  label=' Threshold 2500␣
↪Particles')

ax.set(xlabel='N', ylabel='n',
       title='Dependence of samplesize (n) on microplastic content (P),␣
↪errormargin (e), and total number of fragments (N)');
#plt.legend(bbox_to_anchor=(0., -0.2, 1., -.102), loc='lower left',ncol=2,␣
↪mode="expand", borderaxespad=0.)
plt.legend();

#save your plot
plt.savefig('Sampling_error_random_model.png')
# End of TODO
# -----------------------------------------------------------
```

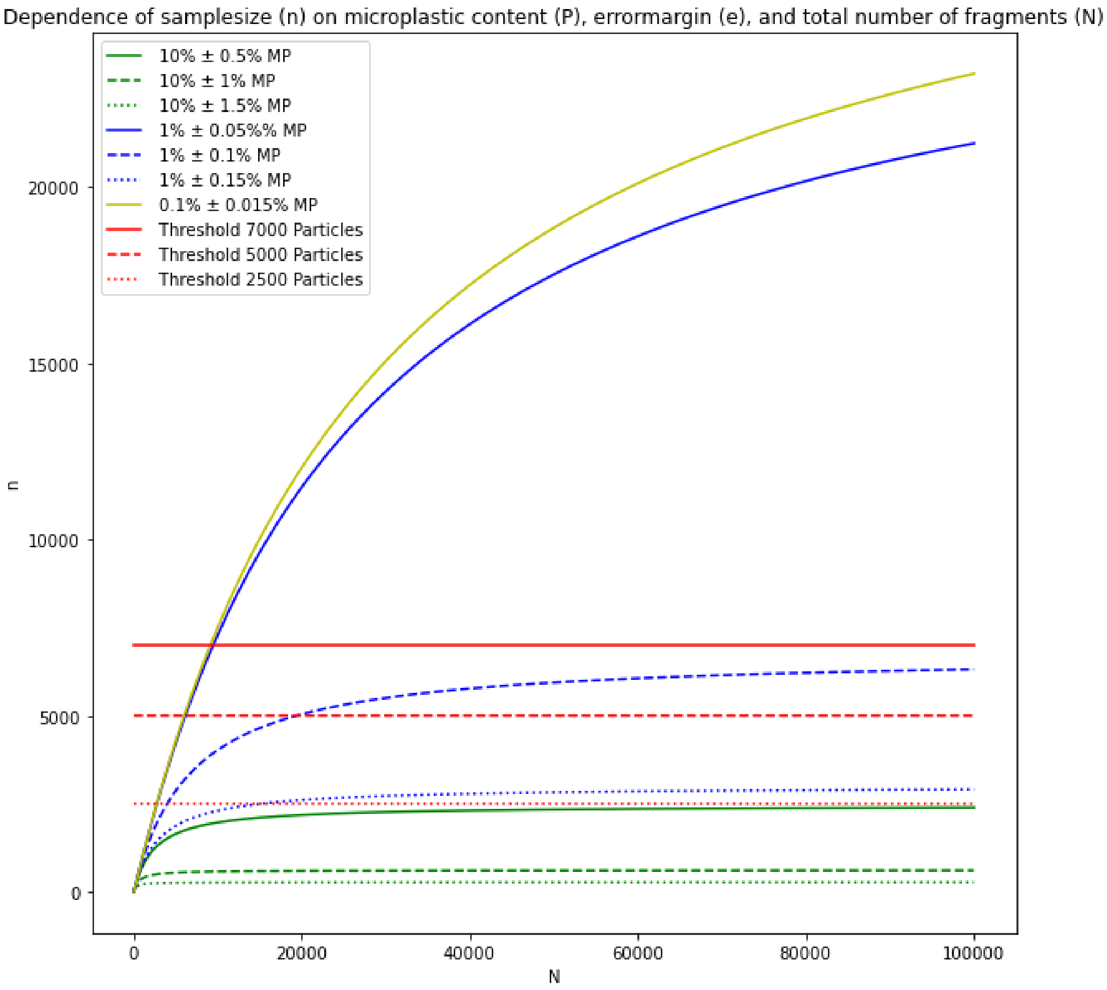

Supplement: Supplementary file 2 — (PDF 379 kb) [file 216_2021_3498_MOESM2_ESM.pdf]
